# Supplementary material for: Benchmarking, detection, and genotyping of structural variants in a population of whole-genome assemblies using the SVGAP pipeline
Source: bioRxiv. 2025 Feb 8:2025.02.07.637096. Preprint. [Version 1] doi: 10.1101/2025.02.07.637096 (PMC11839052; doi:10.1101/2025.02.07.637096)
Supplement: Supplement 1 [file media-1.pdf]

Supplementary Material for

“Benchmarking, detection, and genotyping of structural variants in a population of whole-genome assemblies using the SVGAP pipeline”

Ming Hu[1,6], Penglong Wan[1,6], Chengjie Chen[2,6], Shuyuan Tang[1], Jiahao Chen[1], Liang Wang[1], Mahul Chakraborty[3], Yongfeng Zhou[2], Jinfeng Chen[4], Brandon S. Gaut[5], J.J. Emerson[5,†] & Yi Liao[1,†]

Contents

1. Supplementary Notes
2. Supplementary Methods
3. Supplementary Tables
4. Supplementary Figures
5. References

Supplementary Notes

Supplementary Note 1: A practical guide to using SVGAP

Section 1: Preparing the genome assemblies

Assembly quality requirement

The SVGAP pipeline was originally designed for genome assemblies of chromosome-level quality; however, it can also be effectively applied to contig-level assemblies. Theoretically, there are no specific quality requirements for the genome assembly, such as N50 or contig numbers. Nevertheless, we highly recommend that users include only chromosomes and long contigs when preparing the input genome assemblies. Currently, SVGAP is optimized for haploid genomes. If you have phased genomes with multiple haplotypes, the recommended approach is to treat each haplotype as an individual assembly.

Naming

To effectively utilize the SVGAP pipeline, users must adhere to specific naming guidelines for genome assemblies and their chromosomes/contigs. Here are some key points to keep in mind:

1) Assembly names do not need to include file extensions such as ".fasta" or ".fa".

2) Avoid naming assemblies solely using numbers. For example, naming an assembly "9311" could potentially cause issues in certain situations. Instead, consider using "N9311".

3) Do not name the query assembly in a way that completely matches or overlaps with the names of reference assemblies. For instance, if the reference name is "MH63", it would be advisable to avoid naming any query assembly "H63" or "MH6".

4) Chromosome names should follow the format "GenomeID.ChromosomeID", such as "MH63.chr01".

Splitting (Optional)

If you have an extreme size of plant genomes, such as maize and pepper, you may need to split the genome into small pieces, and then conduct the pairwise genome alignment. Here we provide a script called ***0_SplitFa.pl*** to facilitate this splitting work.

You just need to split the query one in the genome assemblies folder, here is an example:

```
$ perl 0_SplitFa.pl ZH97 2000000 400000
```

This script will split a FASTA file into fixed-sized windows with a specified step size. The first parameter "ZH97" refers to the input FASTA file, the second parameter "2000000" indicates the size of the fixed-sized windows, and the third parameter "400000" denotes the step size for moving along the sequence.

Section 2: a step-by-step tutorial

In this section, we used 49 rice genomes to demonstrate how to use SVGAP step by step:

Constructing a working folder

To start the project, we first constructed a folder named rice:

```
$ mkdir rice
$ mkdir rice/genome
```

Moving working genomes to subfolder

Once the working genomes have been named and filtered for short contigs, they are moved to the rice/genome subfolder. Please be note that even if you split the query genomes, the rice/genome should only contain unsplit files.

Step 0: Whole genome alignment

Currently, SVGAP supports alignment results from a variety of aligners, including Lastz, Last, MUMmer, minimap2, GSAlign, and AnchorWave:

1) For small and medium-sized genomes (<500 Mb), we recommend utilizing Last and MUMmer.

2) For larger genomes, particularly in the case of plants, it is advisable to use minimap2 or AnchorWave instead.

3) If you opt for AnchorWave, please ensure that a dependable gene annotation GFF3 file is accessible for the reference genome and that there are no considerable rearrangements between the reference and query genomes.

In the case of our rice data, we opted to employ MUMmer4 (with default parameters) to conduct alignments between the reference genome (MH63) and each of the 48 query genomes.

Here's an example:

```
$ nucmer -t 4 --prefix=MH63vsBasm ./genome/MH63 ./genome/Basm
```

If you would like to use other alternative aligners, you may refer the following commands:

minimap2

```
$ minimap2 -c --cs=long ./genome/MH63 ./genome/Basm > MH63vsBasm.chr.paf
```

last

```
$ lastdb -P4 -uNEAR MH63dat ./genome/MH63
$ last-train -P4 --revsym -E0.05 -C2 MH63dat ./genome/Basm > MH63vsBasm.train
$ lastal -p MH63vsBasm.train MH63dat Basm > MH63vsBasm.maf
```

Step 1: Converting the alignment output from a particular aligner to the AXT format alignment files

SVGAP incorporates a series of UCSC programs (e.g., axtChain, ChainNet, and netSyntenic developed by Jim Kent) to preprocess alignment files before identifying genomic variants. Hence, it is necessary to convert alignment files from other aligners with different formats, such as *.delta* from MUMmer4, *.paf* from minimap2, and *.maf* from LAST/LASTZ/AnchorWave, to the AXT alignment format (the *.axt* format is described in http://genome.ucsc.edu/goldenPath/help/axt.html) prior to processing them within the pipeline. To facilitate this conversion, SVGAP provides a script called ***1_Convert2Axt.pl*** to handle this task. To find the detailed usage information about the *1_Convert2Axt.pl* script, you can run:

```
$ perl 1_Convert2Axt.pl --help
```

In our case of rice data, we saved all *delta* alignment files produced by MUMmer4 to a single output folder rice/alignment and created a working folder rice/chainnet/Target_Ref.

Rearrange output files

```
$ mkdir  ./rice/alignment
$ mkdir ./rice/chainnet
$ mv *.delta ./rice/alignment
```

Run 1_Convert2Axt.pl

```
$ perl 1_Convert2Axt.pl -ali mummer -input ./rice/alignment -wk ./rice/chainnet
```

Please note that if you split query genomes, you should provide a genome size file (tab-delimited form) named "genomeID.sizes" in the working folder where the *.axt* file will be saved before running *1_Convert2Axt.pl*.

Here is an example of the genome size file:

```
MH63.chr01	23513712
MH63.chr02	32079331
```

After running the above commands, the resulting .axt files have been saved in the rice/chainnet folder and ready for further processing.

Step 2: Constructing chains and nets from pairwise alignments

To accurately identify genetic variants between the reference and query genomes, the raw alignments (.axt file) must be processed for synteny, and paralogous regions need to be removed. This ensures that only the orthologous regions are retained for analysis. This task can be accomplished by employing a set of UCSC programs (find necessary information from http://genomewiki.ucsc.edu/index.php/Whole_genome_alignmen_howto) that are sequentially invoked in the script 2_ChainNetSyn.pl within the SVGAP pipeline which will generate a filtered net file for syntenic alignments only and a single-coverage (on the reference genome) collection of pairwise alignments.

To find more detailed usage about 2_ChainNetSyn.pl script, run:

```
$ perl 2_ChainNetSyn.pl --help
```

In our case we run the command as following:

```
$ perl 2_ChainNetSyn.pl --gd ../genome/ --ad chainnet/Target_Ref/ --lst ../genome/g.lst --sing
```

The filtered *.net* files for syntenic alignments will be saved in ./rice/synnet/ and the sing-coverage alignment *.maf* files will be saved in ./rice/singRaw/.

In most cases, especially when comparing two highly divergent genomes, it is recommended to utilize the additional script ***3_SynNetFilter.pl*** to filter the syntenic alignment *.net* files.This script enhances precision while slightly reducing sensitivity, resulting in improved accuracy for such comparisons.

To see how to use the 3_SynNetFilter.pl script, run:

```
$ perl 3_SynNetFilter.pl --help
```

We run the following command for the rice test data:

```
$ perl 3_SynNetFilter.pl --synnet synnet/ --gd ../genome/ --chain chainnet/Target_Ref
```

The final filtered *.net* files for syntenic alignments are saved in ./rice/CleanSynNet/ which will be used for SV calling and the cleaned single-coverage alignment *.maf* files are saved in ./rice/filtered_sing/ which will be used for SNP calling and SV genotyping.

Step 3: Identification of structural variants (SVs)

The SVGAP pipeline infers SVs directly from the final filtered *net* files (the *net* format is described in http://genome.ucsc.edu/goldenPath/help/net.html). Currently, five common SV types between pairwise genome comparisons can be routinely identified using the *script* ***4_PairwiseSV.pl*** within the SVGAP pipeline, including insertions, deletions, tandem duplications, inversions, and translations. Additionally, local highly divergent regions (i.e. with gaps on both target and query sequences) are defined as a complex type.

To see how to use the *4_PairwiseSV.pl* script, you can run:

```
$ perl 4_PairwiseSV.pl --help
```

Below is the command we run for the rice data:

```
$ perl 4_PairwiseSV.pl --syndir ./rice/CleanSynNet --gd ./rice/genome/ --outdir ./rice/CleanSV
```

The detected SV calls are stored in the directory ./rice/CleanSV/. For each pairwise comparison, two sets of SVs are reported, with coordinates that correspond to both the reference and query genomes.

Step 4: Merge and remove redundant SV calls

Since SVs are identified between the reference genome and each of the query genomes, it is essential to merge the SVs from all comparisons. This merging process aims to generate a non-redundant dataset of SV calls that encompasses all the comparisons. By doing so, each SV can be further genotyped across all individuals involved in the study, enabling construction of the pan-genome. The script ***5_Combined.pl*** within the SVGAP pipeline can work for this task.

To see how to use the *5_Combined.pl* script, you can run:

```
$ perl 5_Combined.pl --help
```

We run the following command to generate the non-redundant SV calls for all 48 pairwise companions.

```
$ perl 5_Combined.pl --sv ./rice/CleanSV --refname MH63
```

Executing the above command will generate a folder called ./rice/CombinedSV/. Within this folder, five types of non-redundant SVs will be generated. It is worth mentioning that the *insertions* and *deletions* have been categorized into two classes according to their lengths; specifically, events that are equal to or longer than 50 bp (>50 bp) are in one class, while the rest are in another.

Step 5: Re-genotyping the merged non-redundant SV calls across all samples based on pairwise (single coverage) alignments

Although the above step has provided preliminary genotyping information, the information still lacks completeness. For instance, the detection of SVs was limited to specific individuals, leaving uncertainty as to whether the absence of these SVs in other individuals is genuine or merely a result of inaccurate sequencing and assembling, or sequence loss in those regions. Therefore, additional methods are necessary to achieve complete genotyping information for all SVs across all samples. The SVGAP utilizes pairwise single coverage alignment files to enhance the genotyping of each SV by extracting and comparing the local alignment in order to determine the SV status in each sample. The scripts ***6_DELGenotyping.pl*** and ***7_INSGenotyping.pl*** within the SVGAP pipeline are utilized to perform genotyping for deletions and insertions (with a length greater than or equal to 50 base pairs), respectively.

Genotyping Deletions (≥50 bp)

To see how to use the 6_DELGenotyping.pl script, you can run:

```
$ perl 6_DELGenoptyping.pl --help
```

We run the tested rice data with the following command:

```
$ perl 6_DELGenoptyping.pl --del ./rice/CombinedSV/All.DELs.50bplarge.bed.combined.sorted.txt \
--singmaf ./rice/filtered_sing/ --refseq ./rice/genome/MH63 --refsize ./rice/genome/MH63.sizes \
--refname MH63 -t 12
```

This step will result in a *vcf* file named All.DELs.50bplarge.bed.combined.sorted.txt.vcf in the folder where

the All.DELs.50bplarge.bed.combined.sorted.txt file is kept.

Genotyping Insertions (≥50 bp)

To see how to use the *7_INSGenotyping.pl* script, you can run:

```
$ perl 7_INSGenotyping.pl --help
```

We run the tested rice data with the following command:

```
$ perl 7_INSGenoptyping.pl --ins ./rice/CombinedSV/All.INTs.50bplarge.bed.combined.sorted.txt \
--query ../genome/ -sing filtered_sing/ --stretcher ../../SV-GAPS/pub/Stretcher/ --refseq ../genome/MH63 \
--refsize ../genome/MH63.sizes --refname MH63
```

This step will result in a *vcf* file named All.INTs.50bplarge.bed.combined.sorted.txt.vcf in the folder where the All.INTs.50bplarge.bed.combined.sorted.txt file is kept.

Genotyping InDels

Small deletions and insertions (<50 bp) are separately genotyped with *8_InDelGenotyping.pl*.

To see how to use the *8_InDelGenotyping.pl* script, you can run:

```
$ perl 8_InDelGenotyping.pl --help
```

Genotyping small deletions in our case:

```
$ perl 8_InDelGenoptyping.pl --indel ./rice/CombinedSV/All.DELs.50bpsmall.bed.combined.sorted.txt \
--indeltype del --refseq ./rice/genome/MH63 --refsize ./rice/genome/MH63.sizes --refname MH63 \
--query ./rice/genome/ --singmaf ./rice/filtered_sing/ --outdir MH63_del --t 12
```

This step will result in a *vcf* file named All.DELs.50bpsmall.bed.combined.sorted.txt.vcf in the folder where the All.DELs.50bpsmall.bed.combined.sorted.txt file is kept.

Genotyping small insertions in our case:

```
$ perl 8_InDelGenoptyping.pl --indel ./rice/CombinedSV/All.INTs.50bpsmall.bed.combined.sorted.txt \
--indeltype ins --refseq  ./rice/genome/MH63 --refsize ./rice/genome/MH63.sizes --refname MH63 \
--query ./rice/genome/ --singmaf ./rice/filtered_sing/ --outdir MH63_ins --t 12
```

This step will result in a *vcf* file named All.INTs.50bpsmall.bed.combined.sorted.txt.vcf in the folder where the All.INTs.50bpsmall.bed.combined.sorted.txt file is kept.

Genotyping SNPs

SVGAP also offers a script called 9_SNPgenotyping.pl, which allows for the direct calling of SNPs from the single-coverage *.maf* files. It generates a *.vcf* file that includes all samples and their respective SNP information.

To see how to use the *9_SNPgenotyping.pl* script, you can run:

```
$ perl 9_SNPgenotyping.pl --help
```

The command we used for the test data is:

```
$ perl 9_SNPgenotyping.pl --refseq ./rice/genome/MH63 --refsize ./rice/genome/MH63.sizes \
--refname MH63 --singmaf ../rice/filtered_sing/ --outdir SNPVCF --t 8
```

The SNP genotyped file All.SNP.vcf will report in the SNPVCF folder.

Step 6: Annotating structural variants

When we acquire precise catalogs of structural variants, it is crucial to elucidate the potential mechanisms driving the formation of SVs and their functional impacts. We offer a script called ***10_SVannotation.pl*** to infer the primary common factors in plant genomes, such as tandem duplication, inverted tandem duplication, transposon elements (TEs) insertion, nest TEs insertion, and complete/partial gene duplication, that may be involved in SV formation.

To see how to use the *10_SVannotation.pl* script, you can run:

```
$ perl 10_SVannotation.pl --help
```

The command we used for the test data is:

```
$ perl 10_SVannotation.pl --ref ./rice/genome/MH63 --vcf All.INTs.50bplarge.bed.combined.sorted.txt.vcf \
--svtype INS --te TE.EDTA.lab.fa --cds MSU7.cds.fa --out INS.annotated.vcf
$ perl 10_SVannotation.pl --ref ./rice/genome/MH63 --vcf All.DELs.50bplarge.bed.combined.sorted.txt.vcf \
--svtype DEL --te TE.EDTA.lab.fa --cds MSU7.cds.fa --out INS.annotated.vcf
```

The annotated *.vcf* file(s) will report in the current folder.

Supplementary Note 2: Evaluation of execution time and memory usage for different genome aligners

Installation of genome aligners

We selected 14 genome aligners to evaluate their execution time and memory usage for plant genome alignment, including minimap2, AnchorWave, MUMmer4, Blasr, BWA-MEN, GraphMap, Last, Lastz, MECAT2, Ngmlr, Pbmm2, GSAlign, Unimap and winnowmap. We installed most of them through ***conda*** with one exception.

Thirteen of them can be installed with conda

```
$ conda install -c bioconda minimap2
$ conda install -c bioconda -c conda-forge anchorwave
$ conda install bioconda::mummer4
$ conda install bioconda::blasr
$ conda install -c bioconda bwa
$ conda install bioconda::graphmap
$ conda install bioconda::last
$ conda install bioconda::lastz
$ conda install ngmlr -c bioconda
$ conda install -c bioconda pbmm2
$ conda install bioconda::gsalign
$ conda install bioconda::unimap
$ conda install bioconda::winnowmap

MECAT2 was installed from GitHub :
$ git clone https://github.com/xiaochuanle/MECAT2.git
$ cd MECAT2
$ make
```

Testing for genome alignment

The command we used for the test data is:

1. **minimap2** ***(version 2.22-r1101)***

## (2min 42s, 7.64 GB)

```
$ time minimap2 -x asm5 ~/MH63RS3.fasta ~/ZS97RS3.fasta > map.paf
```

2. ***AnchorWave (v1.2.3)***

## Extract CDS (0.20 min, 959.61 Mb)

```
$ time anchorwave gff2seq -i MH63RS3.gff3 -r MH63RS3.fasta -o cds.fa
```

## Align CDS to the reference genome (3.20 min, 12.85 Gb)

```
$ time minimap2 -x splice -t 10 -k 12 -a -p 0.4 -N 20 MH63RS3.fasta cds.fa > ref.sam
```

## Align CDS to the query genome (3.53 min, 13.47 Gb)

```
$ time minimap2 -x splice -t 10 -k 12 -a -p 0.4 -N 20 S97RS3.fasta cds.fa > query.sam
```

## Perform genome alignment (78.52 min, 17.13 Gb)

```
$ time anchorwave genoAli -i MH63RS3.gff3 -as cds.fa -r MH63RS3.fasta -a query.sam -ar ref.sam \
-s ZS97RS3.fasta -n SZ.anchors -o anchorwave.maf -f anchorwave.f.maf > SZ.log
```

3. MUMmer4 (4.0.0rc1)

The command we used for the test data is:

## Alignment (103.85 min,4.65 Gb)

```
$ time nucmer -p out MH63RS3.fasta ZS97RS3.fasta
```

4. Blasr (5.3.5-5.3.5)

Blasr is used to align PacBio reads to the reference genome, but it keeps throwing errors during usage, and the cause is currently unclear.

The command we used for the test data is:

```
$ time blasr ZS97RS3.fasta MH63RS3.fasta --out test.out -m 5 --header --nproc 50
```

We tried modifying the parameters, but the errors persist:

```
$ time blasr ZS97RS3.fasta MH63RS3.fasta --out test.out --maxMatch 10000000000 --maxLCPLength 10000000000
```

5. BWA-MEM (0.7.17-r1188)

The command we used for the test data is:

## Construct genome index (4.22 min, 569.72 Gb)

```
$ time bwa index MH63RS3.fasta
```

## Alignment (time-consuming, 10.69 Gb)

```
$ time bwa mem MH63RS3.fasta ZS97RS3.fasta > output.sam
```

6. GraphMap (v0.6.3)

GraphMap is used to align Nanopore reads to the reference genome. The command we used for the test data is:

```
$ time graphmap2 align -r MH63RS3.fasta -d ZS97RS3.fasta -o graphmap_out.sam
```

Unfortunately, the result cannot be output due to our limited memory.

7. ***Last (1584)***

The command we used for the test data is:

### Simple version

## Construct reference genome database (2.07 min, 1.89 Gb)

```
$ time lastdb MHdb MH63RS3.fasta
```

## Alignment (97.40 min, 3.44 Gb)

```
$ time lastal MHdb ZS97RS3.fasta > myalns.maf
```

## Make a dotplot (64.25 min, 118.68 Gb)

```
$ time last-dotplot myalns.maf
```

### Detailed version

## Construct reference genome database (0.48 min,1.53 GB)

```
$ time lastdb -P8 -c -uRY128 MH63db MH63RS3.fasta
```

## Training (0.07 min, 651 Mb)

```
$ time last-train -P8 --revsym -C2 MH63db ZS97RS3.fasta > zs.train
```

## Mang to one alignment (1.17 min,13.99 GB)

```
$ time lastal -P8 -D1e9 -C2 --split-f=MAF+ -p zs.train MH63db ZS97RS3.fasta > many-to-one.maf
```

One to one filtration *(0.40 min, 2.31 GB)*

```
$ time last-split -r many-to-one.maf > one-to-one.maf
```

## Make a dotplot (0.20 min, 98.39 Mb)

```
$ time last-dotplot one-to-one.maf
```

8. ***Lastz (1.04.22)***

LastZ only supports pairwise alignment of one chromosome to another, so we used a looping method for whole genome alignment:

## Extract each chromosome to a new FASTA file

```
$ awk '/^>/ {if (f) close(f); f = "MH63" substr($0, 2,5) ".fa"; print > f} !/^>/ {print > f}' MH63RS3.fasta
$ awk '/^>/ {if (f) close(f); f = "ZS" substr($0, 2,5) ".fa"; print > f} !/^>/ {print > f}' ZS97RS3.fasta
```

## "Looping" alignment (8.47 min, 162.79 Mb)

```
$ for file in ZS*; do name="${file:2:5}"; lastz "MH63${name}.fa" "${file}" --notransition --step=20 --nogapped \
--format=maf > "${name}.maf"; done
```

9. MECAT2 (2.0.0)

MECAT2 is used to align SMRT reads to the reference genome. We used 80 threads because the execution was too slow:

## Alignment (45.47 min, 21.28 Gb)

```
$ time mecat2map -num_threads 80 ZS97RS3.fasta MH63RS3.fasta > result.m4
```

10. Ngmlr (0.2.7)

Ngmlr is also used to align SMRT reads to the reference genome.

The command we used for the test data is:

## Alignment (time-consuming)

```
$ time ngmlr -r ~/MH63RS3.fasta -q ~/ZS97RS3.fasta -o test.sam
```

11. Pbmm2 (1.14.99)

Pbmm2 is also used to align SMRT reads to the reference genome. We also used 80 threads because the execution was too slow:

## Construct genome index (0.10 min, 2.39 Gb)

```
$ time pbmm2 index MH63RS3.fasta ref.mmi
```

## Alignment (57.88 min,438.5 Gb)

```
$ time pbmm2 align ref.mmi ZS97RS3.fasta ref.movie.bam -j 80
```

12. GSAlign (v1.0.22)

The command we used for the test data is:

## Alignment (5.45 min, 2.46 Gb)

```
$ time GSAlign -r MH63RS3.fasta -q ZS97RS3.fasta -o output
```

13. *Unimap (0.1-r41)*

The command we used for the test data is:

### Index-free genome alignment (2.28 min,9.28 Gb)

```
$ time unimap -b24 MH63RS3.fasta ZS97RS3.fasta > out.paf
```

### Index-depended genome alignment

## Construct genome index (0.67 min, 7.31 Gb)

```
$ time unimap -b24 -d out.umi ~/MH63RS3.fasta
```

## Alignment (2.23 min,6.21 Gb)

```
$ time unimap -c out.umi ~/ZS97RS3.fasta > out1.paf
```

14. *winnowmap (2.03)*

The command we used for the test data is:

## Meryl count（1.82 min,2.21 Gb）

```
$ time meryl count k=19 output merylDB MH63RS3.fasta
```

## Meryl print (0.12 min, 1.08 Gb)

```
$ time meryl print greater-than distinct=0.9998 merylDB > repetitive_k19.txt
```

## Alignment (6.45 min, 6.44 Gb)

```
$ time winnowmap -W repetitive_k19.txt -x asm20 MH63RS3.fasta ZS97RS3.fasta > output.paf
```

Supplementary Methods

Approaches for benchmarking analysis using a phylogeny-based simulated dataset

To evaluate the extent to which sequence divergence impacts SV detection, we propose the following strategy for benchmark analysis. ***First***, we selected eight divergent genomes, using one as the reference, from both the *Oryza* and *Solanum* phylogenies. The genomes exhibit varying levels of sequence divergence relative to the reference. ***Second***, for each phylogeny-based genome set, we simulated SVs, including insertions and deletions, in all eight genomes using RSVSim [1]. ***Third***, we identified SVs between the reference and the eight simulated genomes, as well as between the reference and the eight original genomes. ***Fourth***, the latter SV set (background calls) was subtracted from the former to obtain the set of induced SVs. ***Finally***, these remaining SV calls were compared against the simulated SV set to measure the recall, precision, and *F1*-score for each genome.

Step 1: Simulating SVs in the genomes

We used the following script, run_RSVSim.Rscript, to simulate SVs for each genome.

```
#!/usr/bin/env Rscript
library(argparser, quietly = TRUE)
## Create a parser
p <- arg_parser("RSVSim runner")
## Add command line arguments
p <- add_argument(p, "--genome", help = "genome_path", type = "character")
p <- add_argument(p, "--output", help = "output_path", type = "character")
p <- add_argument(p, "--delnum", help = "delnum", type = "character")
p <- add_argument(p, "--insnum", help = "insnum", type = "character")
## Parse the command line arguments
argv <- parse_args(p)
genome_file <- argv$genome
output_file <- argv$output
delnum <- as.numeric(argv$delnum)
insnum <- as.numeric(argv$insnum)
library("RSVSim")
## Setting SV size for DELs and INTs
delSizes = estimateSVSizes(n = delnum, minSize = 50, maxSize = 20000, default = "deletions", hist = TRUE)
intSizes = estimateSVSizes(n = insnum, minSize = 50, maxSize = 20000, default = "insertions", hist = TRUE)
## With additional breakpoint mutations
simulateSV(output = output_file, genome = genome_file, sizeDels = delSizes, dels = delnum, sizeIns = intSizes, ins = insnum,
percCopiedIns = 1)
```

For example, we used the following command to introduce 10,000 deletions and 10,00 insertions into the CJ14 genome.

```
$ Rscript run_RSVSim.Rscripts --genome CJ14 --output CJ14_Simulated –delnum 10000 --insnum 10000
```

The output are files recording the variant's coordinates: **deletions.csv** and **insertions.csv**, and the simulated genome: **genome_rearranged.fasta**, all have been saved in the folder CJ14_Simulated.

Since the coordinates in deletions.csv and insertions.csv are based on the original genomes, they need to be lifted over to the simulated genomes for comparisons between the reference and the simulated genomes. The following commands and the script SimulationReformatSV.pl were used for this purpose.

```
$ cat deletions.csv | grep -v Name | cut -f1-5 > simulatedA.DELs.bed
$ cat insertions.csv | grep -v Name | cut -f1,5-8 > simulatedA.INTs.bed
$ cat simulatedA.DELs.bed simulatedA.INTs.bed | sort -k2,2 -k3,3n > simulatedA.All.variant.sort.bed
$ perl SimulationReformatSV.pl simulatedA.All.variant.sort.bed
```

This step will generate .bed files containing SV coordinates based on the simulated genomes.

Step 2: Whole genome alignment

The generated simulated data can be used for benchmarking aligners and callers. Here we show how we benchmark callers with SVGAP, including Last, MUMmer4, minimap2 and AnchorWave.

```
### LAST
## Construct reference genome database
$ lastdb -P8 -uNEAR Adatabase ./genome/A
## Training
$ last-train -P8 -E0.05 --revsym -C2 Adatabase ./genome/B > AvsB.train
## Alignment
$ lastal --split-f=MAF -p AvsB.train Adatabase ./genome/B > AvsB.chr.maf

### MUMmer4
$ nucmer -t 4 --prefix=AvsB ./genome/A .genome/B

### minimap2
$ minimap2 -t 2 -c --cs=long -x asm5 ./genome/A ./genome/B > AvsB.chr.paf

### AnchorWave
## Extract CDS
$ anchorwave gff2seq -i ./genome/A.gff -r ./genome/A -o ./genome/A.CDS.fa
## Align CDS to the query genome
$ minimap2 -x splice -t 10 -k 12 -a -p 0.4 -N 20 ./genome/B ./genome/A.CDS.fa > B.sam
## Align CDS to the reference genome
$ minimap2 -x splice -t 10 -k 12 -a -p 0.4 -N 20 ./genome/A ./genome/A.CDS.fa > A.sam
## Perform genome alignment
$ anchorwave genoAli -i ./genome/A.gff -as ./genome/A.CDS.fa -r ./genome/A -a B.sam -ar A.sam -s ./genome/B -n B.anchors \
-o B.maf -f AvsB.maf > B.log
```

Step 3: Calling SVs

Using the alignment results from the four tested aligners, we applied the SVGAP pipeline to call SVs between each genome and the reference. The single-coverage alignment *.maf* files (saved in the singRaw directory) and the detected SV calls (stored in the CleanSV directory) were used for subsequent analysis.

We used the following command to calculate the alignable proportion of each genome relative to the reference.

```
$ grep -v '#' singRaw/A.simulatedB.sing.maf | awk -v term="simulatedB" '$2 ~ term' | \
awk '{sum +=$4} END {print  sum}' > AvssimulatedB.alignedLength

## Calculate the total genome size
$ seqkit faidx genome/simulated
$ awk '{sum +=$2} END{print sum}' simulatedB.fai > simulatedB.totallength

$ echo "$(cat AvssimulatedB.alignedLength)/$(cat simulatedB.totallength)" | bc -l | \
awk -v prefix="mappingrate=" '{print prefix $0}'
```

Step 4 : Benchmarking

We calculated recall, precision, and F1 score using the following formula:

```
Precision = TP / (TP + FP)
Recall = TP / (TP + FN)
F1 Score = (2 * Precision * Recall) / (Precision + Recall)
```

, where TP, FP, FN and TN are defined as following:

# TP (True positive) SVGAP predicted there is a SV and it actually has.

# FP (False positive): SVGAP predicted there is a SV but it actually hasn't.

# FN (False negative): SV-GAPS predicted there isn't a SV but it actually has.

#TN (True negative): SVGAP predicted there isn't a SV and it actually hasn't.

Benchmark aligners for DEL (deletion) between genome A and simulated genome A'

```
## Select DELs ≥50 bp
$ cat CleanSV/A.simulatedA.rawSV/AvssimulatedA.chain.filter.tnet.synnet.refiltered.synnet.out.indel.sort | grep -v "#" | awk
'$8>49 {print $5"\t"$6"\t"$6+$8}' > AvssimulatedA.DELs.bed
## Calculation for TP
$ bedtools intersect -wa -wb -a simulatedA.All.variant.sort.bed.del.bed -b AvssimulatedA.DELs.bed -f 0.8 -F 0.8 | cut -f1-3 |
sort | uniq | wc -l | awk -v prefix="TP=" '{print prefix $0}'
## Calculation for FP
$ bedtools intersect -b simulatedA.All.variant.sort.bed.del.bed -a AvssimulatedA.DELs.bed -f 0.8 -F 0.8 -v | sort | uniq | wc -l |
awk -v prefix="FP=" '{print prefix $0}'
## Calculation for FN
$ bedtools intersect -a simulatedA.All.variant.sort.bed.del.bed -b AvssimulatedA.DELs.bed -f 0.8 -F 0.8 -v | sort | uniq | wc -l |
awk -v prefix="FN=" '{print prefix $0}'
```

Benchmark aligners for INT (insertion) between genome A and simulated genome A'

```
## Select INTs ≥50 bp
$ cat CleanSV/simulatedA.A.rawSV/AvssimulatedA.chain.filter.qnet.synnet.refiltered.synn et.out.indel.sort | awk '$8>49' \
> AvssimulatedA.INTs.bed
## Calculation for TP
$ bedtools intersect -wa -wb -a simulatedA.All.variant.sort.bed.ins.bed -b AvssimulatedA.INTs.bed -f 0.8 -F 0.8 | cut -f1-3 | \
```

```
sort | uniq | wc -l | awk -v prefix="TP=" '{print prefix $0}'
## Calculation for FP
$ bedtools intersect -b simulatedA.All.variant.sort.bed.ins.bed -a AvssimulatedA.INTs.bed -f 0.8 -F 0.8 -v | sort | uniq | wc -l | \
awk -v prefix="FP=" '{print prefix $0}'
## Calculation for FN
$ bedtools intersect -a simulatedA.All.variant.sort.bed.ins.bed -b AvssimulatedA.INTs.bed -f 0.8 -F 0.8 -v | sort | uniq | wc -l | \
awk -v prefix="FN=" '{print prefix $0}'
```

Benchmark aligners for DEL (deletion) between genome A and simulated genome B'

```
## Select DELs ≥ 50 bp
$ awk '$8>49' CleanSV/A.B.rawSV/AvsB.chain.filter.tnet.synnet.refiltered.synnet.out.indel.sort > AvsB.DELs.bed
$ awk '$8>49' CleanSV/A.simulatedB.rawSV/AvssimulatedB.chain.filter.tnet.synnet.refiltered.synnet.out.indel.sort \
> AvssimulatedB.DELs.bed
## Extract the different DELs set between A vs. B and A vs. simulatedB
$ bedtools intersect -a AvssimulatedB.DELs.bed -b AvsB.DELs.bed -v -f 0.8 -F 0.8 | awk '{print $5"\t"$6"\t"$6+$8}' \
> simulatedBvsB.diff.DELs.bed
## Calculation for TP
$ bedtools intersect -wa -wb -a simulatedB.All.variant.sort.bed.del.bed -b simulatedBvsB.diff.DELs.bed -f 0.8 -F 0.8 | \
cut -f1-3 | sort | uniq | wc -l | awk -v prefix="TP=" '{print prefix $0}'
## Calculation for FP
$ bedtools intersect -b simulatedB.All.variant.sort.bed.del.bed -a simulatedBvsB.diff.DELs.bed -f 0.8 -F 0.8 -v | sort | uniq | \
wc -l | awk -v prefix="FP=" '{print prefix $0}'
## Calculation for FN
$ bedtools intersect -a simulatedB.All.variant.sort.bed.del.bed -b simulatedBvsB.diff.DELs.bed -f 0.8 -F 0.8 -v | sort | uniq | \
wc -l | awk -v prefix="FN=" '{print prefix $0}'
```

Benchmark aligners for INT (insertion) between genome A and simulated genome B'

```
## Select INTs ≥50 bp
$ grep -v '#' CleanSV/B.A.rawSV/AvsB.chain.filter.qnet.synnet.refiltered.synnet.indel.sort | \
awk '$8>49 {print $5"\t"$6"\t"$6+$8"\t"$1"\t"$2"\t"$3}' > AvsB.INTs.bed
$ grep -v '#' CleanSV/simulatedB.A.rawSV/AvssimulatedB.chain.filter.qnet.synnet.refiltered.synnet.indel.sort | \
awk '$8>49 {print $5"\t"$6"\t"$6+$8"\t"$1"\t"$2"\t"$3}' > AvssimulatedB.INTs.bed
## Extract the different INTs set between A vs. B and A vs. simulatedB
$ bedtools intersect -a AvssimulatedB.INTs.bed -b AvsB.INTs.bed -v -f 0.8 -F 0.8 | cut -f4-6 > simulatedBvsB.diff.INTs.bed
## Calculation for TP
$ bedtools intersect -a simulatedB.All.variant.sort.bed.ins.bed -b simulatedBvsB.diff.INTs.bed -f 0.8 -F 0.8 -wa -wb | \
cut -f1-3 | sort | uniq | wc -l | awk -v prefix="TP=" '{print prefix $0}'
## Calculation for FP
$ bedtools intersect -b simulatedB.All.variant.sort.bed.ins.bed -a simulatedBvsB.diff.INTs.bed -f 0.8 -F 0.8 -v | sort | uniq | \
wc -l | awk -v prefix="FP=" '{print prefix $0}'
## Calculation for FN
$ bedtools intersect -a simulatedB.All.variant.sort.bed.ins.bed -b simulatedBvsB.diff.INTs.bed -f 0.8 -F 0.8 -v | sort | uniq | \
wc -l | awk -v prefix="FN=" '{print prefix $0}'
```

Step 5: Normalization

We used the alignable proportion to normalize the metrics, ensuring that the precision, recall, and F1-score are calculated only from the alignable regions. These metrics were further adjusted using the genome alignment rate, as described below:

$average\_AlignmentRate = \; / \; N_{(The\ number\ of\ aligners)}$

$adjustTP = TP \, / \, average\_AlignmentRate$

$adjustFP = FP * average\_AlignmentRate$

$adjustFN = N_{(SV\ number)} - adjustTP$

Supplementary Tables

Supplementary Table 1: Species selected for testing and comparison of aligners in whole genome alignment of plant genomes.

Species	Genome size	Repeat content	Assemblies used	Reference
Fly (*Drosophila melanogaster)*	~140 Mb	22 %	iso-1 *versus* A4	[2–4]
Human (*Homo sapiens*)	~3.1 Gb	~54 %	CHM13 *versus* GRCh38	[5,6]
Rice (*Oryza sativa*)	~396 Mb	~46 %	MH63 *versus* ZS97	[7]
Tomato (*Solanum lycopersicum*)	~782 Mb	71.77 %	SL4.0 *versus* M82	[8,9]
Maize (*Zea mays*)	~2.2 Gb	~85 %	B73 *versus* Nc350	[10]
Pepper (*Capsicum annuum*)	~3.1 Gb	84.71 %	Ca59 *versus* Zunla-1	[11,12]

Supplementary Table 2: Parameters and commands used for different aligners to evaluate whole genome alignments across genomes of varying complexity.

Aligner	Version	Parameters	Reference
lastz	1.04.22	lastz $REF[multiple] $QRY --format=axt --output=REFvsQRY.axt	Harris 2007
last	1406	lastdb -P8 -uNEAR REFdat $REF	[13]
		lastal REFdat QRY > REFvsQRY.maf	
MUMmer	v4	nucmer --prefix=REFvsQRY.mm $REF $QRY	[14]
		nucmer --maxmatch --prefix=REFvsQRY.max $REF $QRY	
minimap2	2.24-r1122	minimap2 -c --cs=long -x asm5 $REF $QRY > REFvsQRY.asm5.paf	[15]
		minimap2 -c --cs=long -x asm10 $REF $QRY > REFvsQRY.asm10.paf	
		minimap2 -c --cs=long -x asm20 $REF $QRY > REFvsQRY.asm20.paf	
		minimap2 -c --cs=long $REF $QRY > REFvsQRY.def.paf	
AnchorWave	v1.0.1/ v1.2.3	anchorwave genoAli -i REF.gff3 -as cds.fa -r $REF -a cds.sam -ar REF.sam -s $QRY -n	[16]
		anchors -o anchorwave.maf -f anchorwave.f.maf -v SV.vcf -m 0 -t 4	
		anchorwave proali -i REF.gff3 -as cds.fa -r $REF -a cds.sam -ar REF.sam -s $QRY -n	
		anchors	
GSAlign	v1.0.22	-o anchorwave.maf -f anchorwave.f.maf -R 1 -Q 1 -m 0 -t 4	[17]
		GSAlign -r $REF -q $QRY -o REFvsQRY	

Supplementary Table 3: Metrics for evaluating different aligners on whole genome alignments across genomes of varying complexity.

Species	Compared genomes	Lastz default	Last default	MUMmer4 default	MUMmer4 maxmatch	minimap2 default	minimap2 asm5	minimap2 asm10	minimap2 asm20	AnchorWave genoAli	AnchorWave proali	GSAlign
**Execution time (CPU-time hh:mm:ss)**												
Fly	ISO1 versus A4	3:28:03	0:16:05	0:10:17	1:11:33	0:04:44	0:03:55	0:02:45	0:04:43	1:04:03	1:13:33	0:02:07
Rice	MH63 versus ZH97	28:35:04	2:47:24	6:54:34	489:37:29	1:22:07	0:22:20	0:19:43	0:41:09	5:48:20	3:44:18	0:07:58
Tomato	SL4.0 versus M82	> 2 weeks	7:27:13	7:58:48	NA	4:00:07	0:23:49	0:22:05	0:41:22	6:29:18	1:29:57	0:14:53
Maize	B73 versus Nc350	NA	48:07:32	147:10:12	NA	164:18:02	7:50:44	7:15:59	13:38:19	91:38:35	85:39:06	1:14:12
Pepper	CA59 versus Zunla-1	NA	45:52:15	29:58:14	NA	23:09:53	2:40:44	2:38:05	5:06:50	NA	81:05:16	1:05:57
Human	CHM13 versus GRCh38	NA	29:28:26	17:23:35	NA	16:20:02	0:43:04	0:42:11	1:17:29	91:28:32	55:02:40	1:13:54
**Peak memory usage (Gb)**												
Fly	ISO1 versus A4	0.95	1.18	2.79	8.34	4.65	4.004	3.992	4.43	117.8	90.153	1.17
Rice	MH63 versus ZH97	2.91	5.76	7.63	457.4	28	10.84	10.83	19.49	145.7	116.06	2.65
Tomato	SL4.0 versus M82	10.3	8.75	10.78	NA	55.9	15.15	15.02	26.1	96.1	58.57	4.4
Maize	B73 versus Nc350	NA	38.8	30.921	NA	280.5	38.42	36.62	66	202.2	200.78	14.19
Pepper	CA59 versus Zunla-1	NA	41.32	50.61	NA	64.49	16.17	15.92	28.97	NA	203.31	15
Human	CHM13 versus GRCh38	NA	31.48	48.3	NA	147.41	25.68	25.72	36.6	210.04	209.6	14.63
**Number/storage space (Gb) of preliminary alignment blocks**												
Fly	ISO1 versus A4	0.49	2.765	0.383	2.24	0.262	0.255	0.258	0.26	0.266	0.26	0.235
Rice	MH63 versus ZH97	4.38	128.61	5.074	182.99	0.754	0.70	0.714	0.718	0.845	0.826	0.645
Tomato	SL4.0 versus M82	93.17	450.68	8.28	NA	1.49	1.452	1.47	1.48	1.55	1.51	1.38
Maize	B73 versus Nc350	NA	5,020.0	137.00	NA	4.87	3.50	3.834	3.95	5.67	5.6	3.04
Pepper	CA59 versus Zunla-1	NA	3185.29	28.89	NA	6.56	6.41	6.48	6.51	NA	5.20	2.55
Human	CHM13 versus GRCh38	NA	1308.86	18.25	NA	5.56	5.48	5.54	5.63	5.63	5.39	5.19
**Genomic occupancy of the inferred one-to-one orthologous alignments**												
Fly	ISO1 versus A4	0.9522	0.9925	0.9820	0.9926	0.9500	0.9394	0.9434	0.9462	0.9337	0.9228	0.9225
Rice	MH63 versus ZH97	0.8728	0.9827	0.9641	0.9769	0.8724	0.8470	0.8553	0.8558	0.8541	0.8498	0.8305
Tomato	SL4.0 versus M82	NA	0.9937	0.9895	NA	0.9615	0.9524	0.9548	0.9543	0.9480	0.9474	0.9326
Maize	B73 versus Nc350	NA	NA	NA	NA	0.6528	0.5578	0.5789	0.5808	0.5883	0.5910	0.6461

Pepper	CA59 versus Zunla-1	NA	NA	NA	NA	0.8905	0.8829	0.8867	0.8873	NA	0.3520	0.4395
Human	CHM13 versus GRCh38	NA	NA	NA	NA	0.9232	0.9192	0.9212	0.9234	0.8385	0.8350	0.8883

Supplementary Table 4: SVs (deletions and insertions) and their overlap across different aligners, as detected by SVGAP between two real genomes within species: rice, *Drosophila*, and tomato.

Aligners	AnchorWave genoAli	AnchorWave proAli	GSAlign	last	lastz	minimap2 asm10	minimap2 asm20	minimap2 asm5	minimap2 default	MUMmer4 maxmatch	MUMmer4 default
**The number of deletions (≥ 50 bp) detected between two rice genomes: MH63 and ZS97.**											
AnchorWave_genoAli	11,642	9,777	3,187	8,094	7,205	6,196	6,646	5,178	7,512	6,856	6,684
AnchorWave_proAli	9,777	10,800	3,096	7,711	6,937	5,937	6,386	5,001	7,250	6,666	6,516
GSAlign	3,187	3,096	3,735	3,064	2,820	2,870	2,912	2,645	3,067	2,951	3,028
last	8,094	7,711	3,064	10,757	6,896	6,371	6,648	5,447	7,481	7,105	6,825
lastz	7,205	6,937	2,820	6,896	9,600	5,630	5,986	4,790	6,809	5,960	5,955
minimap2_asm10	6,196	5,937	2,870	6,371	5,630	8,118	7,447	6,520	7,238	6,093	6,050
minimap2_asm20	6,646	6,386	2,912	6,648	5,986	7,447	8,860	6,319	7,776	6,309	6,232
minimap2_asm5	5,178	5,001	2,645	5,447	4,790	6,520	6,319	6,683	6,147	5,417	5,409
minmap2_default	7,512	7,250	3,067	7,481	6,809	7,238	7,776	6,147	10,427	6,908	6,850
MUMmer4_max	6,856	6,666	2,951	7,105	5,960	6,093	6,309	5,417	6,908	8,969	7,310
MUMmer4_mm	6,684	6,516	3,028	6,825	5,955	6,050	6,232	5,409	6,850	7,310	7,970
**Overlap of deletion calls (≥ 50 bp) across different aligners, as detected by SVGAP between two rice genomes: MH63 and ZS97.**											
AnchorWave_genoAli	1.0000	0.8398	0.2738	0.6952	0.6189	0.5322	0.5709	0.4448	0.6452	0.5889	0.5741
AnchorWave_proAli	0.9053	1.0000	0.2867	0.7140	0.6423	0.5497	0.5913	0.4631	0.6713	0.6172	0.6033
GSAlign	0.8533	0.8289	1.0000	0.8203	0.7550	0.7684	0.7797	0.7082	0.2840	0.2732	0.8107
last	0.7524	0.7168	0.2848	1.0000	0.6411	0.5923	0.6180	0.5064	0.6955	0.6605	0.6345
lastz	0.7505	0.7226	0.2938	0.7183	1.0000	0.5865	0.6235	0.4990	0.7093	0.6208	0.6203
minimap2_asm10	0.7632	0.7313	0.3535	0.7848	0.6935	1.0000	0.9173	0.8032	0.8916	0.7506	0.7453
minimap2_asm20	0.7501	0.7208	0.3287	0.7503	0.6756	0.8405	1.0000	0.7132	0.8777	0.7121	0.7034
minimap2_asm5	0.7748	0.7483	0.3958	0.8151	0.7167	0.9756	0.9455	1.0000	0.9198	0.8106	0.8094
minmap2_default	0.7204	0.6953	0.2941	0.7175	0.6530	0.6942	0.7458	0.5895	1.0000	0.6625	0.6569
MUMmer4_max	0.7644	0.7432	0.3290	0.7922	0.6645	0.6793	0.7034	0.6040	0.7702	1.0000	0.8150
MUMmer4_mm	0.8386	0.8176	0.3799	0.8563	0.7472	0.7591	0.7819	0.6787	0.8595	0.9172	1.0000
**The number of insertions (≥ 50 bp) detected between two rice genomes: MH63 and ZS97.**											

AnchorWave_genoAli	11,542	9,899	3,124	8,169	7,400	6,269	6,696	5,226	7,471	6,877	6,723
AnchorWave_proAli	9,899	10,879	3,067	7,810	7,158	6,048	6,486	5,099	7,250	6,763	6,626
GSAlign	3,124	3,067	3,821	3,176	2,891	3,047	3,092	2,825	3,229	3,068	3,120
last	8,169	7,810	3,176	10,484	7,084	6,509	6,801	5,543	7,514	7,181	6,914
lastz	7,400	7,158	2,891	7,084	9,860	5,813	6,211	4,908	6,864	6,202	6,092
minimap2_asm10	6,269	6,048	3,047	6,509	5,813	8,213	7,584	6,599	7,342	6,227	6,197
minimap2_asm20	6,696	6,486	3,092	6,801	6,211	7,584	8,891	6,397	7,849	6,416	6,384
minimap2_asm5	5,226	5,099	2,825	5,543	4,908	6,599	6,397	6,708	6,222	5,515	5,527
minmap2_default	7,471	7,250	3,229	7,514	6,864	7,342	7,849	6,222	10,076	6,954	6,929
MUMmer4_max	6,877	6,763	3,068	7,181	6,202	6,227	6,416	5,515	6,954	8,665	7,533
MUMmer4_mm	6,723	6,626	3,120	6,914	6,092	6,197	6,384	5,527	6,929	7,533	8,097

Overlap of insertion calls (≥ 50 bp) between different aligners, as detected by SVGAP between two rice genomes: MH63 and ZS97.

AnchorWave_genoAli	1.0000	0.8577	0.2707	0.7078	0.6411	0.5431	0.5801	0.4528	0.6473	0.5958	0.5825
AnchorWave_proAli	0.9099	1.0000	0.2819	0.7179	0.6580	0.5559	0.5962	0.4687	0.6664	0.6217	0.6091
GSAlign	0.8176	0.8027	1.0000	0.8312	0.7566	0.7974	0.8092	0.7393	0.8451	0.8029	0.8165
last	0.7792	0.7449	0.3029	1.0000	0.6757	0.6209	0.6487	0.5287	0.7167	0.6849	0.6595
lastz	0.7505	0.7260	0.2932	0.7185	1.0000	0.5896	0.6299	0.4978	0.6961	0.6290	0.6178
minimap2_asm10	0.7633	0.7364	0.3710	0.7925	0.7078	1.0000	0.9234	0.8035	0.8939	0.7582	0.7545
minimap2_asm20	0.7531	0.7295	0.3478	0.7649	0.6986	0.8530	1.0000	0.7195	0.8828	0.7216	0.7180
minimap2_asm5	0.7791	0.7601	0.4211	0.8263	0.7317	0.9838	0.9536	1.0000	0.9275	0.8222	0.8239
minmap2_default	0.7415	0.7195	0.3205	0.7457	0.6812	0.7287	0.7790	0.6175	1.0000	0.6902	0.6877
MUMmer4_max	0.7937	0.7805	0.3541	0.8287	0.7158	0.7186	0.7405	0.6365	0.8025	1.0000	0.8694
MUMmer4_mm	0.8303	0.8183	0.3853	0.8539	0.7524	0.7653	0.7884	0.6826	0.8557	0.9303	1.0000

The number of deletions (≥ 50 bp) detected between two *Drosophila* genomes: iso-1 and A4.

AnchorWave_genoAli	2,203	1,930	819	1,742	1,603	1,766	1,812	1,551	1,777	1,609	1,546
AnchorWave_proAli	1,930	1,999	786	1,632	1,529	1,649	1,686	1,479	1,670	1,544	1,501
GSAlign	819	786	984	823	791	836	833	784	840	804	792
last	1,742	1,632	823	2,214	1,694	1,853	1,840	1,667	1,816	1,788	1,646

lastz	1,603	1,529	791	1,694	2,156	1,661	1,682	1,495	1,670	1,568	1,497
minimap2_asm10	1,766	1,649	836	1,853	1,661	2,199	2,061	1,864	1,951	1,728	1,645
minimap2_asm20	1,812	1,686	833	1,840	1,682	2,061	2,248	1,808	2,019	1,735	1,645
minimap2_asm5	1,551	1,479	784	1,667	1,495	1,864	1,808	1,905	1,747	1,629	1,552
minmap2_default	1,777	1,670	840	1,816	1,670	1,951	2,019	1,747	2,230	1,724	1,647
MUMmer4_max	1,609	1,544	804	1,788	1,568	1,728	1,735	1,629	1,724	1,987	1,738
MUMmer4_mm	1,546	1,501	792	1,646	1,497	1,645	1,645	1,552	1,647	1,738	1,819
**Overlap of deletion calls (≥ 50 bp) between different aligners, as detected by SVGAP between two *Drosophila* genomes: iso-1 and A4.**											
AnchorWave_genoAli	1.0000	0.8761	0.3718	0.7907	0.7276	0.8016	0.8225	0.7040	0.8066	0.7304	0.7018
AnchorWave_proAli	0.9655	1.0000	0.3932	0.8164	0.7649	0.8249	0.8434	0.7399	0.8354	0.7724	0.7509
GSAlign	0.8323	0.7988	1.0000	0.8364	0.8039	0.8496	0.8465	0.7967	0.8537	0.8171	0.8049
last	0.7868	0.7371	0.3717	1.0000	0.7651	0.8369	0.8311	0.7529	0.8202	0.8076	0.7435
lastz	0.7435	0.7092	0.3669	0.7857	1.0000	0.7704	0.7801	0.6934	0.7746	0.7273	0.6943
minimap2_asm10	0.8031	0.7499	0.3802	0.8427	0.7553	1.0000	0.9372	0.8477	0.8872	0.7858	0.7481
minimap2_asm20	0.8060	0.7500	0.3706	0.8185	0.7482	0.9168	1.0000	0.8043	0.8981	0.7718	0.7318
minimap2_asm5	0.8142	0.7764	0.4115	0.8751	0.7848	0.9785	0.9491	1.0000	0.9171	0.8551	0.8147
minmap2_default	0.7969	0.7489	0.3767	0.8143	0.7489	0.8749	0.9054	0.7834	1.0000	0.7731	0.7386
MUMmer4_max	0.8098	0.7771	0.4046	0.8998	0.7891	0.8697	0.8732	0.8198	0.8676	1.0000	0.8747
MUMmer4_mm	0.8499	0.8252	0.4354	0.9049	0.8230	0.9043	0.9043	0.8532	0.9054	0.9555	1.0000
**The number of insertions (≥ 50 bp) detected between two Drosophila genomes: iso-1 and A4.**											
AnchorWave_genoAli	2,185	1,914	766	1,737	1,609	1,752	1,778	1,543	1,758	1,595	1,533
AnchorWave_proAli	1,914	1,999	726	1,623	1,525	1,628	1,659	1,453	1,654	1,527	1,481
GSAlign	766	726	957	808	767	810	810	755	810	776	778
last	1,737	1,623	808	2,456	1,715	1,831	1,834	1,638	1,812	1,837	1,671
lastz	1,609	1,525	767	1,715	2,245	1,683	1,715	1,494	1,699	1,574	1,509
minimap2_asm10	1,752	1,628	810	1,831	1,683	2,273	2,088	1,895	1,960	1,692	1,614
minimap2_asm20	1,778	1,659	810	1,834	1,715	2,088	2,326	1,819	2,019	1,703	1,623
minimap2_asm5	1,543	1,453	755	1,638	1,494	1,895	1,819	1,958	1,736	1,554	1,500

minmap2_default	1,758	1,654	810	1,812	1,699	1,960	2,019	1,736	2,349	1,681	1,618
MUMmer4_max	1,595	1,527	776	1,837	1,574	1,692	1,703	1,554	1,681	2,171	1,745
MUMmer4_mm	1,533	1,481	778	1,671	1,509	1,614	1,623	1,500	1,618	1,745	1,908
**Overlap of insertion calls (≥ 50 bp) between different aligners, as detected by SVGAP between two *Drosophila* genomes: iso-1 and A4.**											
AnchorWave_genoAli	1.0000	0.8760	0.3506	0.7950	0.7364	0.8018	0.8137	0.7062	0.8046	0.7300	0.7016
AnchorWave_proAli	0.9575	1.0000	0.3632	0.8119	0.7629	0.8144	0.8299	0.7269	0.8274	0.7639	0.7409
GSAlign	0.8004	0.7586	1.0000	0.8443	0.8015	0.8464	0.8464	0.7889	0.8464	0.8109	0.8130
last	0.7072	0.6608	0.3290	1.0000	0.6983	0.7455	0.7467	0.6669	0.7378	0.7480	0.6804
lastz	0.7167	0.6793	0.3416	0.7639	1.0000	0.7497	0.7639	0.6655	0.7568	0.7011	0.6722
minimap2_asm10	0.7708	0.7162	0.3564	0.8055	0.7404	1.0000	0.9186	0.8337	0.8623	0.7444	0.7101
minimap2_asm20	0.7644	0.7132	0.3482	0.7885	0.7373	0.8977	1.0000	0.7820	0.8680	0.7322	0.6978
minimap2_asm5	0.7880	0.7421	0.3856	0.8366	0.7630	0.9678	0.9290	1.0000	0.8866	0.7937	0.7661
minmap2_default	0.7484	0.7041	0.3448	0.7714	0.7233	0.8344	0.8595	0.7390	1.0000	0.7156	0.6888
MUMmer4_max	0.7347	0.7034	0.3574	0.8462	0.7250	0.7794	0.7844	0.7158	0.7743	1.0000	0.8038
MUMmer4_mm	0.8035	0.7762	0.4078	0.8758	0.7909	0.8459	0.8506	0.7862	0.8480	0.9146	1.0000
**The number of deletions (≥ 50 bp) detected between two tomato genomes: SL4 and M82.**											
AnchorWave_genoAli	4,654	4,036	1,160	2,448	93	2,600	2,766	2,263	2,631	NA	2,209
AnchorWave_proAli	4,036	4,398	1,144	2,381	91	2,491	2,660	2,198	2,548	NA	2,178
GSAlign	1,160	1,144	1,504	1,124	35	1,159	1,154	1,106	1,157	NA	1,138
last	2,448	2,381	1,124	4,135	104	2,511	2,517	2,266	2,520	NA	2,363
lastz	93	91	35	104	213	114	121	94	122	NA	85
minimap2_asm10	2,600	2,491	1,159	2,511	114	4,540	4,112	3,533	3,809	NA	2,450
minimap2_asm20	2,766	2,660	1,154	2,517	121	4,112	5,020	3,406	4,210	NA	2,475
minimap2_asm5	2,263	2,198	1,106	2,266	94	3,533	3,406	3,618	3,205	NA	2,280
minmap2_default	2,631	2,548	1,157	2,520	122	3,809	4,210	3,205	5,398	NA	2,483
MUMmer4_default	2,209	2,178	1,138	2,363	85	2,450	2,475	2,280	2,483	NA	3,083
**Overlap of deletion calls (≥ 50 bp) between different aligners, as detected by SVGAP between two tomato genomes: SL4 and M82.**											
AnchorWave_genoAli	1.0000	0.8672	0.2492	0.5260	0.0200	0.5587	0.5943	0.4862	0.5653	NA	0.4746

AnchorWave_proAli	0.9177	1.0000	0.2601	0.5414	0.0207	0.5664	0.6048	0.4998	0.5794	NA	0.4952
GSAlign	0.7713	0.7606	1.0000	0.7473	0.0233	0.7706	0.7673	0.7354	0.7693	NA	0.7566
last	0.5920	0.5758	0.2718	1.0000	0.0252	0.6073	0.6087	0.5480	0.6094	NA	0.5715
lastz	0.4366	0.4272	0.1643	0.4883	1.0000	0.5352	0.5681	0.4413	0.5728	NA	0.3991
minimap2_asm10	0.5727	0.5487	0.2553	0.5531	0.0251	1.0000	0.9057	0.7782	0.8390	NA	0.5396
minimap2_asm20	0.5510	0.5299	0.2299	0.5014	0.0241	0.8191	1.0000	0.6785	0.8386	NA	0.4930
minimap2_asm5	0.6255	0.6075	0.3057	0.6263	0.0260	0.9765	0.9414	1.0000	0.8858	NA	0.6302
minmap2_default	0.4874	0.4720	0.2143	0.4668	0.0226	0.7056	0.7799	0.5937	1.0000	NA	0.4600
MUMmer4_default	0.7165	0.7065	0.3691	0.7665	0.0276	0.7947	0.8028	0.7395	0.8054	NA	1.0000
**The number of insertions (≥ 50 bp) detected between two tomato genomes: SL4 and M82.**											
AnchorWave_genoAli	4,814	4,282	1,309	3,209	98	3,108	3,229	2,829	3,228	NA	2,934
AnchorWave_proAli	4,282	4,588	1,287	3,109	92	3,011	3,120	2,769	3,136	NA	2,900
GSAlign	1,309	1,287	1,754	1,429	46	1,441	1,461	1,385	1,457	NA	1,426
last	3,209	3,109	1,429	6,100	125	3,256	3,290	3,045	3,331	NA	3,159
lastz	98	92	46	125	194	103	110	85	106	NA	89
minimap2_asm10	3,108	3,011	1,441	3,256	103	4,223	3,941	3,644	3,709	NA	3,161
minimap2_asm20	3,229	3,120	1,461	3,290	110	3,941	4,562	3,538	3,865	NA	3,199
minimap2_asm5	2,829	2,769	1,385	3,045	85	3,644	3,538	3,713	3,378	NA	3,008
minmap2_default	3,228	3,136	1,457	3,331	106	3,709	3,865	3,378	5,181	NA	3,238
MUMmer4_default	2,934	2,900	1,426	3,159	89	3,161	3,199	3,008	3,238	NA	3,796
**Overlap of insertion calls (≥ 50 bp) between different aligners, as detected by SVGAP between two tomato genomes: SL4 and M82.**											
AnchorWave_genoAli	1.0000	0.8895	0.2719	0.6666	0.0204	0.6456	0.6708	0.5877	0.6705	NA	0.6095
AnchorWave_proAli	0.9333	1.0000	0.2805	0.6776	0.0201	0.6563	0.6800	0.6035	0.6835	NA	0.6321
GSAlign	0.7463	0.7338	1.0000	0.8147	0.0262	0.8216	0.8330	0.7896	0.8307	NA	0.8130
last	0.5261	0.5097	0.2343	1.0000	0.0205	0.5338	0.5393	0.4992	0.5461	NA	0.5179
lastz	0.5052	0.4742	0.2371	0.6443	1.0000	0.5309	0.5670	0.4381	0.5464	NA	0.4588
minimap2_asm10	0.7360	0.7130	0.3412	0.7710	0.0244	1.0000	0.9332	0.8629	0.8783	NA	0.7485
minimap2_asm20	0.7078	0.6839	0.3203	0.7212	0.0241	0.8639	1.0000	0.7755	0.8472	NA	0.7012

minimap2_asm5	0.7619	0.7458	0.3730	0.8201	0.0229	0.9814	0.9529	1.0000	0.9098	NA	0.8101
minmap2_default	0.6230	0.6053	0.2812	0.6429	0.0205	0.7159	0.7460	0.6520	1.0000	NA	0.6250
MUMmer4_default	0.7729	0.7640	0.3757	0.8322	0.0234	0.8327	0.8427	0.7924	0.8530	NA	1.0000

Supplementary Table 5: Benchmark results with reference-based simulated SVs in tomato, rice, and *Drosophila*.

Aligner	# of total calls	# of accurate calls	Recall (Sensitivity)	Precision	FDR	F1
*Simulated **deletions** in tomato (assembly = SL4; n = 28,440; size ≥ 50 bp)*						
AnchorWave_genoAli	28,060	27,490	0.9666	0.9797	0.0203	0.9731
AnchroWave_proAli	27,693	26,230	0.9223	0.9472	0.0528	0.9346
GSAlign	26,215	26,167	0.9201	0.9982	0.0018	0.9575
**last**	**28,225**	**28,102**	**0.9881**	**0.9956**	**0.0044**	**0.9919**
lastz	21,521	19,909	0.7000	0.9251	0.0749	0.7970
minimap2_asm10	27,562	27,466	0.9658	0.9965	0.0035	0.9809
minimap2_asm20	27,573	27,443	0.9649	0.9953	0.0047	0.9799
minimap2_asm5	27,542	27,452	0.9653	0.9967	0.0033	0.9807
minimap2_default	28,323	27,304	0.9601	0.9640	0.0360	0.9620
MUMmer4_default	27,913	27,857	0.9795	0.9980	0.0020	0.9887
*Simulated **insertions** in tomato (assembly = SL4; n = 15,245; size ≥ 50 bp)*						
AnchorWave_genoAli	15,271	14,594	0.9573	0.9557	0.0443	0.9565
AnchroWave_proAli	15,293	14,318	0.9392	0.9362	0.0638	0.9377
GSAlign	14,387	14,385	0.9436	0.9999	0.0001	0.9709
**last**	**15,137**	**15,098**	**0.9904**	**0.9974**	**0.0026**	**0.9939**
lastz	12,644	11,918	0.7818	0.9426	0.0574	0.8547
minimap2_asm10	14,651	14,627	0.9595	0.9984	0.0016	0.9785
minimap2_asm20	14,662	14,614	0.9586	0.9967	0.0033	0.9773
minimap2_asm5	14,641	14,620	0.9590	0.9986	0.0014	0.9784
minimap2_default	14,931	14,645	0.9606	0.9808	0.0192	0.9706
MUMmer4_default	15,066	15,042	0.9867	0.9984	0.0016	0.9925
*Simulated **deletions** in rice (assembly = Nipponbare; n = 20,328; size ≥ 50 bp)*						
AnchorWave_genoAli	19,805	19,452	0.9569	0.9822	0.0178	0.9694
AnchroWave_proAli	19,086	18,308	0.9006	0.9592	0.0408	0.9290
GSAlign	17,796	17,737	0.8725	0.9967	0.0033	0.9305
last	**19,974**	**19,828**	**0.9754**	**0.9927**	**0.0073**	**0.9840**
lastz	15,648	14,908	0.7334	0.9527	0.0473	0.8288
minimap2_asm10	18,980	18,882	0.9289	0.9948	0.0052	0.9607
minimap2_asm20	18,956	18,830	0.9263	0.9934	0.0066	0.9587

minimap2_asm5	18,941	18,848	0.9272	0.9951	0.0049	0.9599
minimap2_default	19,673	19,197	0.9444	0.9758	0.0242	0.9598
MUMmer4_default	19,344	19,279	0.9484	0.9966	0.0034	0.9719
MUMmer4_maxmatch	19,502	19,359	0.9523	0.9927	0.0073	0.9721
unimap	17,785	17,693	0.8704	0.9948	0.0052	0.9284
winnowmap	15,870	15,801	0.7773	0.9957	0.0043	0.8730
Simulated ***insertions*** *in rice (assembly = Nipponbare; n =* 10,046*; size ≥ 50 bp)*						
AnchorWave_genoAli	9,987	9,641	0.9597	0.9654	0.0346	0.9625
AnchorWave_proAli	9,971	9,384	0.9341	0.9411	0.0589	0.9376
GSAlign	9,350	9,336	0.9293	0.9985	0.0015	0.9627
last	**9,887**	**9,855**	**0.9810**	**0.9968**	**0.0032**	**0.9888**
lastz	8,241	8,088	0.8051	0.9814	0.0186	0.8846
minimap2_asm10	9,249	9,229	0.9187	0.9978	0.0022	0.9566
minimap2_asm20	9,239	9,205	0.9163	0.9963	0.0037	0.9546
minimap2_asm5	9,240	9,226	0.9184	0.9985	0.0015	0.9568
minimap2_default	9,589	9,416	0.9373	0.9820	0.0180	0.9591
MUMmer4_default	9,757	9,744	0.9699	0.9987	0.0013	0.9841
MUMmer4_maxmatch	9,757	9,712	0.9668	0.9954	0.0046	0.9809
unimap	8,470	8,457	0.8418	0.9985	0.0015	0.9135
winnowmap	8,276	8,264	0.8226	0.9986	0.0014	0.9021
Simulated ***deletions*** *in Drosophila (assembly =* iso-1*; n =* 7,264*; size ≥ 50 bp)*						
AnchorWave_genoAli	7,001	6,935	0.9547	0.9906	0.0094	0.9723
AnchorWave_proAli	6,777	6,588	0.9069	0.9721	0.0279	0.9384
GSAlign	6,415	6,399	0.8809	0.9975	0.0025	0.9356
last	**7,200**	**7,125**	**0.9809**	**0.9896**	**0.0104**	**0.9852**
lastz	6,743	6,541	0.9005	0.9700	0.0300	0.9340
minimap2_asm10	6,994	6,951	0.9569	0.9939	0.0061	0.9750
minimap2_asm20	6,981	6,916	0.9521	0.9907	0.0093	0.9710
minimap2_asm5	6,985	6,945	0.9561	0.9943	0.0057	0.9748
minimap2_default	7,112	7,015	0.9657	0.9864	0.0136	0.9759
MUMmer4_default	6,957	6,941	0.9555	0.9977	0.0023	0.9762
MUMmer4_maxmatch	7,117	7,042	0.9694	0.9895	0.0105	0.9793

Simulated ***insertions*** *in Drosophila (assembly* = iso-1*; n* = 4,290*; size ≥ 50 bp)*						
AnchorWave_genoAli	4,156	3,874	0.9030	0.9321	0.0679	0.9174
AnchorWave_proAli	4,099	3,760	0.8765	0.9173	0.0827	0.8964
GSAlign	3,928	3,923	0.9145	0.9987	0.0013	0.9547
last	**4,246**	**4,211**	**0.9816**	**0.9918**	**0.0082**	**0.9866**
lastz	4,083	3,978	0.9273	0.9743	0.0257	0.9502
minimap2_asm10	4,099	4,070	0.9487	0.9929	0.0071	0.9703
minimap2_asm20	4,080	4,039	0.9415	0.9900	0.0100	0.9651
minimap2_asm5	4,086	4,066	0.9478	0.9951	0.0049	0.9709
minimap2_default	4,171	4,062	0.9469	0.9739	0.0261	0.9602
MUMmer4_default	4,165	4,154	0.9683	0.9974	0.0026	0.9826
MUMmer4_maxmatch	4,230	4,198	0.9786	0.9924	0.0076	0.9854

Supplementary Table 6: Benchmark results across aligners and callers with phylogeny-based simulated SVs in rice and tomato.

Rice	MH63	R498	9311	ZS97	Nipp	Oruf	CJ14	Opun
Recall without normalization for simulated 10,000 deletions in different rice genomes (size ≥ 50 bp)								
minimap2	0.9803	0.8828	0.8266	0.8089	0.6862	0.5645	0.6464	0.1602
MUMmer4	0.976	0.8811	0.8221	0.8035	0.6793	0.5637	0.6342	0.1283
AnchorWave	0.9853	0.8887	0.8343	0.8196	0.6873	0.5785	0.6632	0.1961
last	0.9915	0.8922	0.8384	0.8216	0.6897	0.5786	0.6687	0.205
last-split	0.9869	0.8891	0.837	0.8175	0.6914	0.5791	0.6572	0.1985
Anchorwave	0.9852	0.8888	0.8344	0.8195	0.6868	0.579	0.6622	0.1966
SVIM-asm	0.9711	0.8737	0.8183	0.7992	0.6712	0.5549	0.6324	0.1427
paftools	0.9655	0.8562	0.792	0.7692	0.6093	0.4784	0.5558	0.0168
Assemblytics	0.5934	0.5464	0.5173	0.4988	0.4417	0.3756	0.4149	0.0863
SyRI	0.5857	0.526	0.5089	0.4844	0.4223	0.3584	0.3858	0.0252
GSAlign	0.0018	0.0132	0.0178	0.0222	0.0426	0.0408	0.0527	0.0152
MUM&CO	0.7468	0.6285	0.5597	0.546	0.3715	0.287	0.2378	0
Precision without normalization for simulated 10,000 deletions in different rice genomes (size ≥ 50 bp)								
minimap2	0.9849	0.9540	0.9304	0.8871	0.8078	0.7923	0.7991	0.3518
MUMmer4	0.9950	0.9911	0.9850	0.9807	0.9031	0.9461	0.9573	0.7752
Anchorwave	0.9842	0.9578	0.9369	0.9020	0.8451	0.8410	0.8629	0.5324
last	0.9961	0.9646	0.9100	0.8730	0.8614	0.8784	0.8376	0.5963
last-split	0.9962	0.9821	0.9636	0.9649	0.9283	0.9316	0.9070	0.6803
Anchorwave	0.9822	0.9489	0.9275	0.8885	0.8396	0.8085	0.8337	0.4458
SVIM-asm	0.9720	0.8349	0.8182	0.7624	0.6642	0.5666	0.6130	0.2039
paftools	0.9698	0.9240	0.8979	0.8591	0.7896	0.6706	0.7065	0.0680
Assemblytics	0.8716	0.9476	0.9287	0.9188	0.8613	0.8268	0.8028	0.6426
SyRI	0.9894	0.9781	0.9658	0.9636	0.9302	0.9057	0.8820	0.7500
GSAlign	0.0455	1.0000	1.0000	1.0000	1.0000	1.0000	1.0000	1.0000
MUM&co	0.9624	0.9190	0.8698	0.8370	0.6834	0.5879	0.5141	0.0000
F1 score without normalization for simulated 10,000 deletions in different rice genomes (size ≥ 50 bp)								
minimap2	0.9826	0.9170	0.8755	0.8462	0.7420	0.6593	0.7147	0.2201
MUMmer4	0.9854	0.9329	0.8962	0.8833	0.7754	0.7065	0.7629	0.2202
Anchorwave	0.9848	0.9219	0.8826	0.8588	0.7581	0.6855	0.7500	0.2866
last	0.9938	0.9270	0.8727	0.8465	0.7660	0.6977	0.7437	0.3051
last-split	0.9915	0.9333	0.8959	0.8851	0.7925	0.7142	0.7621	0.3073
Anchorwave	0.9837	0.9178	0.8785	0.8526	0.7556	0.6748	0.7381	0.2729
SVIM-asm	0.9715	0.8538	0.8183	0.7804	0.6677	0.5607	0.6226	0.1679
paftools	0.9676	0.8888	0.8416	0.8116	0.6878	0.5584	0.6222	0.0269
Assemblytics	0.7061	0.6931	0.6645	0.6466	0.5840	0.5165	0.5471	0.1522
SyRI	0.7358	0.6841	0.6666	0.6447	0.5809	0.5136	0.5368	0.0488
GSAlign	0.0035	0.0261	0.0350	0.0434	0.0817	0.0784	0.1001	0.0299
MUM&co	0.8410	0.7465	0.6811	0.6609	0.4813	0.3857	0.3252	0.0000
Recall without normalization for simulated 10,000 insertions in different rice genomes (size ≥ 50 bp)								
minimap2	0.9847	0.9101	0.8679	0.847	0.7714	0.6571	0.7477	0.2795
MUMmer4	0.9945	0.9243	0.8829	0.8662	0.7736	0.6855	0.7781	0.2959

Anchorwave	0.9975	0.9287	0.8842	0.8709	0.7751	0.6897	0.7889	0.3718
last	0.9984	0.9314	0.8897	0.8751	0.7845	0.695	0.7958	0.3991
last-split	0.9969	0.9289	0.8851	0.8698	0.7799	0.6917	0.7851	0.3885
Anchorwave	0.9975	0.9284	0.884	0.8709	0.776	0.6901	0.7905	0.1966
SVIM-asm	0.9716	0.8911	0.8491	0.8201	0.7173	0.6168	0.6991	0.1803
paftools	0.8425	0.7615	0.7169	0.6937	0.5817	0.4834	0.5474	0.023
Assemblytics	0.8499	0.7904	0.7519	0.7286	0.6569	0.5817	0.663	0.1749
SYRI	0.9314	0.8403	0.7909	0.7644	0.6484	0.5512	0.5956	0.0461
GSalign	0.0014	0.0032	0.0034	0.0041	0.0069	0.005	0.0095	0.0036
MUM&co	0.8722	0.7421	0.6849	0.6635	0.5171	0.287	0.3978	0
Precision without normalization for simulated 10,000 insertions in different rice genomes (size ≥ 50 bp)								
minimap2	0.9767	0.9289	0.9086	0.8672	0.7957	0.7392	0.7884	0.4026
MUMmer4	0.9996	0.9940	0.9878	0.9833	0.9716	0.9606	0.9749	0.8950
Anchorwave	0.9909	0.9680	0.9514	0.9301	0.8888	0.8865	0.9099	0.7461
last	0.9990	0.9796	0.9259	0.8982	0.8445	0.9042	0.9193	0.7821
last-split	0.9996	0.9871	0.9721	0.9700	0.9136	0.9401	0.9408	0.8674
Anchorwave	0.9879	0.9614	0.9453	0.9201	0.8927	0.8617	0.8902	0.5175
SVIM-asm	0.9605	0.8427	0.8022	0.7472	0.6294	0.5307	0.6182	0.2079
paftools	0.9536	0.8761	0.8562	0.7928	0.6988	0.6178	0.6833	0.0939
Assemblytics	0.8924	0.9643	0.9529	0.9444	0.9222	0.8914	0.9052	0.9081
SYRI	0.9806	0.9867	0.9793	0.9752	0.9566	0.9435	0.9652	0.9147
GSalign	0.0376	1.0000	1.0000	1.0000	1.0000	1.0000	1.0000	1.0000
MUM&co	0.9726	0.8689	0.8048	0.7622	0.6190	0.5874	0.5449	0.0000
F1 score without normalization for simulated 10,000 insertions in different rice genomes (size ≥ 50 bp)								
minimap2	0.9807	0.9194	0.8878	0.8570	0.7834	0.6957	0.7675	0.3299
MUMmer4	0.9970	0.9579	0.9324	0.9210	0.8614	0.8001	0.8655	0.4448
Anchorwave	0.9942	0.9479	0.9166	0.8995	0.8281	0.7758	0.8451	0.4963
last	0.9987	0.9549	0.9074	0.8865	0.8134	0.7859	0.8531	0.5285
last-split	0.9982	0.9571	0.9266	0.9172	0.8415	0.7970	0.8559	0.5366
Anchorwave	0.9927	0.9446	0.9136	0.8948	0.8303	0.7664	0.8374	0.2849
SVIM-asm	0.9660	0.8662	0.8250	0.7820	0.6705	0.5705	0.6562	0.1931
paftools	0.8946	0.8148	0.7804	0.7399	0.6349	0.5424	0.6079	0.0370
Assemblytics	0.8706	0.8687	0.8405	0.8226	0.7673	0.7040	0.7654	0.2933
SYRI	0.9554	0.9076	0.8751	0.8570	0.7729	0.6959	0.7366	0.0878
GSalign	0.0027	0.0064	0.0068	0.0082	0.0137	0.0100	0.0188	0.0072
MUM&co	0.9197	0.8005	0.7400	0.7094	0.5635	0.3856	0.4599	0.0000

**Tomato**	**SL4**	**M82**	**ZY65**	**ZY56**	**ZY57**	**ZY60**	**ZY61**	**ZY59**
Recall without normalization for simulated ~13,200 deletions in different tomato genomes (size ≥ 50 bp)								
minimap2	0.9594	0.8840	0.9274	0.8060	0.8077	0.4149	0.3080	0.2597
MUMmer4	0.9877	0.9090	0.9572	0.7833	0.8075	0.4001	0.3045	0.2483
AnchorWave	0.9832	0.9113	0.9540	0.7582	0.7831	0.3429	0.2518	0.2248
last-split	0.9935	0.9076	0.9612	0.8177	0.8479	0.4409	0.3299	0.2853
SVIM-asm	0.8780	0.8106	0.8446	0.7431	0.7593	0.4235	0.3149	0.2584
SyRI	0.5277	0.4896	0.5193	0.4775	0.4813	0.2403	0.1615	0.0900

paftools	0.8638	0.7953	0.8312	0.7292	0.7377	0.3593	0.2426	0.1824
Assemblytics	0.4652	0.4161	0.4472	0.3929	0.3905	0.2439	0.1896	0.1454
AnchorWave	0.9831	0.9114	0.9548	0.7583	0.6540	0.3436	0.2526	0.2254
MUM&co	0.8612	0.7052	0.7972	0.4110	0.4328	0.0039	0.0004	0.0001
Precision without normalization for simulated ~13,200 deletions in different tomato genomes (size ≥ 50 bp)								
minimap2	0.9076	0.8532	0.8865	0.8041	0.8220	0.5206	0.4475	0.4092
MUMmer4	0.9962	0.9884	0.9950	0.9708	0.9747	0.8101	0.7350	0.7079
AnchorWave	0.9785	0.8941	0.9638	0.8929	0.9098	0.6805	0.5494	0.5518
last-split	0.9972	0.9820	0.9903	0.9565	0.9663	0.8476	0.7663	0.5646
SVIM-asm	0.7715	0.6842	0.5255	0.3318	0.5873	0.0750	0.0513	0.0396
SyRI	0.9863	0.9642	0.9644	0.9380	0.9438	0.8501	0.7848	0.7428
paftools	0.8363	0.7553	0.7911	0.5464	0.5593	0.3564	0.2914	0.2474
Assemblytics	0.9321	0.9063	0.9091	0.8620	0.8634	0.6228	0.5508	0.4956
AnchorWave	0.9744	0.8717	0.9559	0.8574	0.8716	0.5352	0.4050	0.3919
Mumandco	0.9778	0.9162	0.9645	0.6634	0.7004	0.0584	0.0230	0.0122
F1 score without normalization for simulated ~13,200 deletions in different tomato genomes (size ≥ 50 bp)								
minimap2	0.9328	0.8683	0.9065	0.8051	0.8148	0.4618	0.3649	0.3177
MUMmer4	0.9919	0.9470	0.9757	0.8670	0.8833	0.5357	0.4306	0.3676
AnchorWave	0.9808	0.9026	0.9589	0.8201	0.8417	0.4560	0.3454	0.3195
last-split	0.9953	0.9433	0.9755	0.8816	0.9032	0.5801	0.4613	0.3791
SVIM-asm	0.8213	0.7421	0.6479	0.4587	0.6624	0.1275	0.0882	0.0687
SyRI	0.6875	0.6495	0.6751	0.6329	0.6375	0.3747	0.2679	0.1605
paftools	0.8498	0.7748	0.8107	0.6247	0.6362	0.3579	0.2648	0.2100
Assemblytics	0.6207	0.5703	0.5995	0.5398	0.5377	0.3505	0.2820	0.2249
AnchorWave	0.9787	0.8911	0.9554	0.8048	0.7473	0.4185	0.3111	0.2862
MUM&co	0.9158	0.7969	0.8729	0.5075	0.5350	0.0074	0.0007	0.0002
Recall without normalization for simulated ~14,800 insertions in different tomato genomes (size ≥ 50 bp)								
minimap2	0.9776	0.9289	0.9514	0.8338	0.8611	0.5593	0.4609	0.4038
MUMmer4	0.9961	0.9495	0.9747	0.8375	0.8622	0.5542	0.4567	0.4088
AnchorWave	0.9956	0.9503	0.9738	0.8141	0.8365	0.4545	0.3505	0.3266
last-split	0.9978	0.9468	0.9745	0.8482	0.8826	0.5896	0.4888	0.4396
SVIM-asm	0.9560	0.9088	0.9310	0.8494	0.8672	0.5761	0.4620	0.3958
SyRI	0.9248	0.8560	0.8849	0.7709	0.7803	0.4032	0.2767	0.1672
paftools	0.7428	0.7066	0.7257	0.6635	0.6733	0.4077	0.3068	0.2374
Assemblytics	0.8815	0.8159	0.8415	0.7393	0.7563	0.5271	0.4348	0.3650
anchorwave	0.9959	0.9507	0.9745	0.8151	0.7045	0.4578	0.3533	0.3310
MUM&co	0.8633	0.7472	0.8228	0.5045	0.5184	0.0084	0.0010	0.0005
Precision without normalization for simulated ~14,800 insertions in different tomato genomes (size ≥ 50 bp)								
minimap2	0.9780	0.9129	0.9584	0.8748	0.9009	0.6438	0.5562	0.5238
Mummer4	1.0000	0.9868	0.9952	0.9795	0.9862	0.8742	0.8431	0.8433
AnchorWave	0.9881	0.9185	0.9697	0.9117	0.9296	0.7545	0.6326	0.6698
last-split	0.9998	0.9858	0.9897	0.9628	0.9728	0.9022	0.8611	0.7793
SVIM-asm	0.8623	0.7880	0.7668	0.7213	0.6937	0.3510	0.2748	0.2524
SyRI	0.9921	0.9817	0.9818	0.9675	0.9642	0.9225	0.8561	0.8704
paftools	0.9232	0.8440	0.8885	0.7809	0.6226	0.4349	0.3609	0.3046
Assemblytics	0.9577	0.9430	0.9461	0.9172	0.9211	0.8078	0.7341	0.7296

AnchorWave	0.9860	0.9045	0.9655	0.8788	0.8977	0.6214	0.4984	0.5122
MUM&co	0.9727	0.8756	0.9243	0.6659	0.6943	0.1249	0.0526	0.0825
F1 score without normalization for simulated ~14,800 insertions in different tomato genomes (size ≥ 50 bp)								
minimap2	0.9778	0.9208	0.9548	0.8538	0.8806	0.5986	0.5041	0.4561
MUMmer4	0.9981	0.9678	0.9849	0.9029	0.9200	0.6783	0.5925	0.5507
AnchorWave	0.9919	0.9341	0.9718	0.8601	0.8806	0.5673	0.4511	0.4391
last-split	0.9988	0.9659	0.9821	0.9019	0.9255	0.7132	0.6236	0.5621
SVIM-asm	0.9067	0.8441	0.8410	0.7801	0.7708	0.4362	0.3447	0.3082
SyRI	0.9573	0.9146	0.9309	0.8581	0.8625	0.5612	0.4182	0.2805
paftools	0.8233	0.7692	0.7989	0.7174	0.6470	0.4209	0.3317	0.2668
Assemblytics	0.9180	0.8749	0.8908	0.8187	0.8306	0.6379	0.5461	0.4866
AnchorWave	0.9910	0.9270	0.9700	0.8458	0.7895	0.5272	0.4135	0.4021
MUM&co	0.9148	0.8063	0.8706	0.5741	0.5936	0.0157	0.0020	0.0011

Supplementary Table 7: Commands and parameters for testing different methods to call SVs in plant genomes.

Methods	Aligners	Parameters	Reference
Assemblytics	MUMmer4.0	Assemblytics REFvsQRY.mm.delta REF **100**/500/1000/10000 1 40000<br>Assemblytics REFvsQRY.max.delta REF **100**/500/1000/10000 1 40000	18
SyRI	minimap2	minimap2 -ax asm5 --eqx -t 4 $REF $QRY > REF.QRY.sam<br>syri -c REFvsQRY.sam -r $REF -q $QRY -k -F S	19
	MUMmer4.0	nucmer -t 4 --prefix=REF.QRY.mm $REF $QRY<br>show-coords -THrd REF.QRY.mm.delta > REF.QRY.mm.coords<br>syri -d REFvsQRY.mm.delta -c REFvsQRY4.mm.coords -r $REF -q $QRY	
SVIM-asm	minimap2	minimap2 -a -x asm5 --cs -r2k -t 4 $REF $QRY > REF.QRY.sam<br>samtools sort -m4G -@4 -o REF.QRY.sorted.bam REF.QRY.sam<br>samtools index REF.QRY.sorted.bam<br>svim-asm haploid ./SV REF.QRY.sorted.bam $REF	20
paftools	minimap2	minimap2 -c --cs=long $REF $QRY > REFvsQRY.def.paf	15
GSAlign	GSAlign	GSAlign -r $REF -q $QRY -o REFvsQRY	17
MUM&Co	MUMmer4.0	mumandco_v3.8.sh -r $REF -q $QRY -g genome.size -o REFvsQRY -t 4	21
AnchorWave	AnchorWave	anchorwave genoAli -i REF.gff3 -as cds.fa -r $REF -a cds.sam -ar REF.sam -s $QRY -n anchors -o anchorwave.maf -f anchorwave.f.maf -v SV.vcf -m 0 -t 4	16
SVGAP	minimap2/last	see manual	This study

Supplementary Table 8: Pairwise comparison of SV calls between different methods.

SVs detected between two rice genome assemblies: MH63 and ZS97.

	AnchorWave	Assemblytics	GSAlign	paftools	MUM&co	SVGAP last	SVGAP minimap2	SVIM-asm	SyRI minimap2	SyRI MUMmer4
AnchorWave	**24,960**	10,261	1,399	12,741	6,287	16,207	14,985	9,854	4,482	2,830
Assemblytics	10,249	**11,993**	985	8,566	4,708	9,689	9,832	7,517	3,538	3,159
GSAlign	1,394	985	**2,557**	1,328	152	1,383	1,497	1,208	805	724
paftools	12,741	8,576	1,334	**19,889**	5,541	12,697	16,952	10,912	5,532	2,528
MUM&co	6,280	4,711	152	5,537	**19,514**	5,883	5,893	5,258	2,253	762
SVGAPS/ last	16,213	9,700	1,388	12,700	5,888	**21,241**	15,008	10,198	4,820	2,735
SVGAPS/ minimap2	14,988	9,843	1,503	16,992	5,897	15,008	**20,503**	11,650	5,804	2,812
SVIM-asm	9,849	7,525	1,211	10,905	5,257	10,189	11,640	**13,402**	6,258	2,377
SyRI/ minimap2	4,479	3,544	807	5,531	2,252	4,817	5,802	6,269	**6,571**	1,510
SyRI/ MUMmer4	2,831	3,167	727	2,530	762	2,736	2,814	2,381	1,511	**3,606**

SVs detected between two tomato genome assemblies: SL4 and M82.

	AnchorWave	Assemblytics	GSAlign	paftools	MUM&co	SVGAP last	SVGAP minimap2	SVIM-asm	SyRI minimap2	SyRI MUMmer4
AnchorWave	**10,494**	3,231	703	1,985	5,850	5,600	6,347	4,601	3,455	2,103
Assemblytics	3,229	**4,146**	526	994	3,284	2,958	3,304	3,093	2,413	2,193
GSAlign	702	527	**1,152**	41	809	625	797	765	701	476
MUM&co	1,986	995	41	**4,695**	1,967	2,131	2,173	1,501	1,280	506
paftools	5,842	3,285	810	1,966	**13,170**	5,684	9,772	5,914	4,627	2,113
SVGAP/ last	5,604	2,961	626	2,131	5,691	**8,619**	6,260	4,614	3,499	1,795
SVGAP/ minimap2	6,347	3,306	798	2,173	9,778	6,260	**11,449**	5,883	4,594	2,105
SVIM-asm	4,598	3,096	766	1,499	5,913	4,611	5,879	**7,346**	5,246	2,045
SyRI/ minimap2	3,453	2,414	702	1,279	4,628	3,497	4,592	5,250	**5,435**	1,761
SyRI/ MUMmer4	2,103	2,196	476	506	2,116	1,795	2,105	2,045	1,762	**2,680**

Supplementary Table 9: Recall of the merging step in SVGAP evaluated using population-scale simulation data based on *Oryza* and *Solanum* phylogeny.

**Benchmarking in rice**		**SVs retrieved from the merging step in SVGAP**							
SV types	# of simulated SVs	Nipp	MH63	R498	9311	ZS97	Oruf	CJ14	Opun
Deletions (< 50 bp)	1,266	1,265	894	900	871	906	839	802	208
Deletions (≥ 50 bp)	1,711	1,707	1,265	1,270	1,196	1,245	1,146	1,155	377
Insertions (< 50 bp)	864	798	620	618	596	619	559	546	163
Insertions (≥ 50 bp)	1,368	1,367	1,013	1,019	975	1,012	932	918	390
**Benchmarking in tomato**		**SVs retrieved from the merging step in SVGAP**							
	# of simulated SVs	SL4	M82	ZY65	ZY56	ZY57	ZY60	ZY61	ZY59
Deletions (≥ 50 bp)	3,017	2,978	2,815	2,774	2,755	2,566	1,748	1,406	1,034
Insertions (≥ 50 bp)	3,382	3,356	3,245	3,149	3,036	2,906	1,695	1,340	1,106

Supplementary Table 10: Performance of the genotyping step in SVGAP evaluated using population-scale simulation data based on *Oryza* and *Solanum* phylogeny.

Accuracy, error, and missing rate for benchmarking analysis for insertions using *Oryza* phylogeny-based data.

	Size	Nipp	MH63	R498	9311	ZS97	Oruf	CJ14	Opun	Size	Nipp	MH63	R498	9311	ZS97	Oruf	CJ14	Opun
**# of retrieved calls**		**798**	**620**	**618**	**596**	**619**	**559**	**546**	**163**	**1,367**	**1,013**	**1,019**	**975**	**1,012**	**932**	**918**	**390**	**798**
**Accuracy**																		
SIM0	<50 bp	730	611	614	592	613	557	542	160	≥ 50 bp	1,348	1,012	1,018	974	1,010	929	917	386
SIM1	<50 bp	783	580	586	559	583	515	506	132	≥ 50 bp	1,341	967	982	935	970	887	869	316
SIM2	<50 bp	783	577	579	560	583	518	499	125	≥ 50 bp	1,339	975	985	936	973	879	859	305
SIM3	<50 bp	778	589	588	570	592	526	509	134	≥ 50 bp	1,338	970	985	935	979	889	864	309
SIM4	<50 bp	784	589	596	571	594	530	507	127	≥ 50 bp	1,347	974	983	944	977	872	862	317
SIM5	<50 bp	778	589	593	567	591	528	504	132	≥ 50 bp	1,338	973	982	937	971	880	872	307
SIM6	<50 bp	782	579	583	562	588	520	503	116	≥ 50 bp	1,342	980	988	948	974	891	869	319
SIM7	<50 bp	779	586	591	565	589	528	514	130	≥ 50 bp	1,342	974	982	936	968	888	863	316
SIM8	<50 bp	783	587	591	564	587	516	500	115	≥ 50 bp	1,329	979	987	938	974	886	866	317
SIM9	<50 bp	781	589	594	569	589	516	504	131	≥ 50 bp	1,346	972	986	937	977	885	875	311
SIM10	<50 bp	783	582	580	560	585	519	505	139	≥ 50 bp	1,342	968	979	934	966	876	877	320
SIM11	<50 bp	786	584	586	562	581	525	503	126	≥ 50 bp	1,335	973	985	940	967	881	865	309
SIM12	<50 bp	776	582	586	561	582	523	511	132	≥ 50 bp	1,349	973	981	929	969	882	861	321
SIM13	<50 bp	778	585	590	570	591	524	506	125	≥ 50 bp	1,349	979	992	936	972	890	860	322
SIM14	<50 bp	783	592	590	567	592	528	506	130	≥ 50 bp	1,338	973	983	932	975	887	872	314
SIM15	<50 bp	772	584	588	568	583	522	506	131	≥ 50 bp	1,353	972	982	930	967	881	874	313
SIM16	<50 bp	780	584	587	566	585	524	502	130	≥ 50 bp	1,342	968	983	935	963	878	863	314
SIM17	<50 bp	782	585	591	565	589	524	502	133	≥ 50 bp	1,343	973	987	936	971	891	873	311
SIM18	<50 bp	778	585	589	571	594	514	504	128	≥ 50 bp	1,347	977	987	936	970	877	873	320
SIM19	<50 bp	782	586	586	567	590	523	502	127	≥ 50 bp	1,344	970	980	935	969	886	868	303
SIM20	<50 bp	780	585	589	566	581	518	503	126	≥ 50 bp	1,345	972	989	938	973	884	867	312
**Average**		778	586	589	567	589	524	507	130		1,343	975	986	938	973	886	870	317
**Accuracy**		97.5%	94.5%	95.4%	95.1%	95.1%	93.7%	92.8%	79.7%		98.2%	96.2%	96.8%	96.2%	96.2%	95.0%	94.8%	81.3%

**Error rate**																	
SIM0	<50 bp	35	6	2	1	2	0	0	0	≥ 50 bp	8	0	0	0	0	0	0	1
SIM1	<50 bp	9	18	13	14	14	22	19	9	≥ 50 bp	8	28	20	18	20	24	33	22
SIM2	<50 bp	9	24	21	13	15	18	18	10	≥ 50 bp	8	24	17	22	22	27	36	25
SIM3	<50 bp	15	15	14	7	9	15	12	4	≥ 50 bp	10	27	20	24	20	27	33	23
SIM4	<50 bp	3	18	11	13	13	13	15	7	≥ 50 bp	7	25	22	14	21	39	35	20
SIM5	<50 bp	14	20	14	13	15	17	16	7	≥ 50 bp	7	23	22	20	22	31	32	21
SIM6	<50 bp	9	21	17	15	14	20	17	2	≥ 50 bp	7	20	19	17	25	26	35	4
SIM7	<50 bp	11	20	14	14	16	17	15	8	≥ 50 bp	10	23	17	15	25	23	34	21
SIM8	<50 bp	8	17	13	15	15	21	20	1	≥ 50 bp	12	19	17	21	21	28	35	5
SIM9	<50 bp	12	16	15	14	14	21	19	6	≥ 50 bp	7	21	12	18	18	24	29	22
SIM10	<50 bp	8	21	19	18	16	19	19	6	≥ 50 bp	7	29	21	23	26	38	25	19
SIM11	<50 bp	5	19	15	16	18	18	17	6	≥ 50 bp	12	26	19	21	26	30	33	18
SIM12	<50 bp	11	19	16	16	17	18	13	6	≥ 50 bp	5	23	18	22	22	28	31	15
SIM13	<50 bp	9	19	16	13	14	17	21	6	≥ 50 bp	7	22	14	21	20	25	37	16
SIM14	<50 bp	10	14	16	15	13	13	20	9	≥ 50 bp	10	29	21	25	24	28	31	17
SIM15	<50 bp	15	22	15	11	17	20	17	10	≥ 50 bp	3	28	23	28	27	30	30	23
SIM16	<50 bp	9	19	17	11	15	15	18	8	≥ 50 bp	10	27	21	22	26	35	37	25
SIM17	<50 bp	11	17	13	14	13	15	20	7	≥ 50 bp	5	24	17	22	23	23	27	22
SIM18	<50 bp	7	19	16	11	11	22	20	7	≥ 50 bp	11	20	15	16	22	34	25	19
SIM19	<50 bp	10	18	17	12	11	17	17	8	≥ 50 bp	7	23	19	17	24	29	28	27
SIM20	<50 bp	10	19	14	14	18	22	19	10	≥ 50 bp	7	27	16	23	27	26	33	24
**Average**		11	18	15	13	14	17	17	7		8	23	18	19	22	27	30	19
**Error rate**		1.37%	2.93%	2.37%	2.16%	2.23%	3.07%	3.07%	4.00%		0.59%	2.29%	1.73%	2.00%	2.17%	2.94%	3.31%	4.75%
**Missing rate**																	
SIM0	<50 bp	33	3	2	3	4	2	4	3	≥ 50 bp	11	1	1	1	2	3	1	3
SIM1	<50 bp	6	22	19	23	22	22	21	22	≥ 50 bp	18	18	17	22	22	21	16	52
SIM2	<50 bp	6	19	18	23	21	23	29	28	≥ 50 bp	20	14	17	17	17	26	23	60
SIM3	<50 bp	5	16	16	19	18	18	25	25	≥ 50 bp	19	16	14	16	13	16	21	58
SIM4	<50 bp	11	13	11	12	12	16	24	29	≥ 50 bp	13	14	14	17	14	21	21	53
SIM5	<50 bp	6	11	11	16	13	14	26	24	≥ 50 bp	22	17	15	18	19	21	14	62

SIM6	<50 bp	7	20	18	19	17	19	26	45	≥ 50 bp	18	13	12	10	13	15	14	67
SIM7	<50 bp	8	14	13	17	14	14	17	25	≥ 50 bp	15	16	20	24	19	21	21	53
SIM8	<50 bp	7	16	14	17	17	22	26	47	≥ 50 bp	26	15	15	16	17	18	17	68
SIM9	<50 bp	5	15	9	13	16	22	23	26	≥ 50 bp	14	20	21	20	17	23	14	57
SIM10	<50 bp	7	17	19	18	18	21	22	18	≥ 50 bp	18	16	19	18	20	18	16	51
SIM11	<50 bp	7	17	17	18	20	16	26	31	≥ 50 bp	20	14	15	14	19	21	20	63
SIM12	<50 bp	11	19	16	19	20	18	22	25	≥ 50 bp	13	17	20	24	21	22	26	54
SIM13	<50 bp	11	16	12	13	14	18	19	32	≥ 50 bp	11	12	13	18	20	17	21	52
SIM14	<50 bp	5	14	12	14	14	18	20	24	≥ 50 bp	19	11	15	18	13	17	15	59
SIM15	<50 bp	11	14	15	17	19	17	23	22	≥ 50 bp	11	13	14	17	18	21	14	54
SIM16	<50 bp	9	17	14	19	19	20	26	25	≥ 50 bp	15	18	15	18	23	19	18	51
SIM17	<50 bp	5	18	14	17	17	20	24	23	≥ 50 bp	19	16	15	17	18	18	18	57
SIM18	<50 bp	13	16	13	14	14	23	22	28	≥ 50 bp	9	16	17	23	20	21	20	51
SIM19	<50 bp	6	16	15	17	18	19	27	28	≥ 50 bp	16	20	20	23	19	17	22	60
SIM20	<50 bp	8	16	15	16	20	19	24	27	≥ 50 bp	15	14	14	14	12	22	18	54
Average		9	16	14	16	17	18	23	27		16	15	15	17	17	19	18	54
Missing rate		1.12%	2.53%	2.26%	2.75%	2.67%	3.25%	4.15%	16.25%		1.19%	1.46%	1.51%	1.78%	1.68%	2.03%	1.92%	13.91%

Accuracy, error, and missing rate for benchmarking analysis for deletions using *Oryza* phylogeny-based data.

# of retrieved calls		**1,265**	**894**	**900**	**871**	**906**	**839**	**802**	**208**		**1,707**	**1,265**	**1,270**	**1,196**	**1,245**	**1,146**	**1,155**	**377**
**Accuracy**		**Nipp**	**MH63**	**R498**	**9311**	**ZS97**	**Oruf**	**CJ14**	**Opun**		**Nipp**	**MH63**	**R498**	**9311**	**ZS97**	**Oruf**	**CJ14**	**Opun**
SIM0	<50 bp	1,182	828	832	803	845	768	733	139	≥ 50 bp	1,690	1,264	1,270	1,195	1,243	1,143	1,153	376
SIM1	<50 bp	1,242	825	831	804	847	762	732	146	≥ 50 bp	1,693	1,248	1,257	1,179	1,228	1,125	1,137	329
SIM2	<50 bp	1,251	838	841	812	852	783	737	138	≥ 50 bp	1,700	1,246	1,252	1,175	1,223	1,127	1,133	331
SIM3	<50 bp	1,244	836	841	810	851	769	738	151	≥ 50 bp	1,689	1,247	1,257	1,180	1,225	1,121	1,135	340
SIM4	<50 bp	1,228	828	834	798	841	764	734	145	≥ 50 bp	1,689	1,244	1,252	1,176	1,223	1,125	1,129	335
SIM5	<50 bp	1,242	827	834	799	839	755	733	143	≥ 50 bp	1,691	1,245	1,252	1,172	1,219	1,117	1,134	336
SIM6	<50 bp	1,243	839	841	813	851	774	735	144	≥ 50 bp	1,687	1,248	1,255	1,180	1,223	1,120	1,132	338
SIM7	<50 bp	1,240	839	839	809	848	773	739	148	≥ 50 bp	1,686	1,246	1,254	1,173	1,220	1,121	1,132	342
SIM8	<50 bp	1,241	838	840	810	844	770	733	147	≥ 50 bp	1,685	1,246	1,255	1,184	1,227	1,125	1,142	342
SIM9	<50 bp	1,237	838	845	806	849	760	728	136	≥ 50 bp	1,691	1,244	1,249	1,176	1,215	1,112	1,132	336

SIM10	<50 bp	1,247	825	830	802	842	766	724	142	≥ 50 bp	1,684	1,243	1,253	1,174	1,225	1,121	1,136	340
SIM11	<50 bp	1,248	838	834	814	851	765	730	147	≥ 50 bp	1,691	1,244	1,250	1,167	1,214	1,124	1,132	331
SIM12	<50 bp	1,239	835	836	806	845	769	738	148	≥ 50 bp	1,690	1,250	1,255	1,178	1,224	1,123	1,139	337
SIM13	<50 bp	1,241	833	838	810	849	763	736	139	≥ 50 bp	1,689	1,249	1,258	1,179	1,227	1,124	1,142	336
SIM14	<50 bp	1,245	833	838	804	847	768	737	152	≥ 50 bp	1,693	1,246	1,254	1,172	1,220	1,122	1,135	338
SIM15	<50 bp	1,244	833	835	807	851	770	730	146	≥ 50 bp	1,696	1,247	1,253	1,180	1,225	1,124	1,132	339
SIM16	<50 bp	1,243	829	829	799	838	774	734	144	≥ 50 bp	1,693	1,240	1,252	1,177	1,221	1,119	1,131	344
SIM17	<50 bp	1,238	833	846	806	846	771	735	141	≥ 50 bp	1,684	1,250	1,260	1,180	1,229	1,125	1,135	336
SIM18	<50 bp	1,242	847	856	817	853	771	735	143	≥ 50 bp	1,689	1,239	1,250	1,173	1,212	1,116	1,128	336
SIM19	<50 bp	1,239	829	839	802	843	770	731	146	≥ 50 bp	1,694	1,246	1,256	1,177	1,223	1,124	1,129	334
SIM20	<50 bp	1,241	831	838	803	847	767	731	151	≥ 50 bp	1,685	1,246	1,253	1,176	1,221	1,123	1,133	331
Average		1,239	833	838	806	847	768	733	145		1,690	1,247	1,255	1,177	1,223	1,123	1,135	338
Accuracy		97.94%	93.22%	93.11%	92.58%	93.45%	91.56%	91.46%	69.51%		99.00%	98.54%	98.79%	98.44%	98.25%	97.98%	98.25%	89.77%
**Error rate**																		
SIM0	<50 bp	1	0	0	0	0	0	0	0	≥ 50 bp	7	0	0	1	1	0	0	0
SIM1	<50 bp	1	2	2	1	1	2	2	0	≥ 50 bp	8	9	7	6	8	12	9	7
SIM2	<50 bp	0	2	1	1	1	1	1	0	≥ 50 bp	3	9	10	8	11	10	13	15
SIM3	<50 bp	0	2	1	0	0	1	1	0	≥ 50 bp	11	10	8	8	10	16	9	6
SIM4	<50 bp	1	2	1	1	0	1	1	0	≥ 50 bp	11	11	10	7	10	14	16	9
SIM5	<50 bp	2	2	1	2	1	1	1	0	≥ 50 bp	11	9	10	8	14	17	13	9
SIM6	<50 bp	0	1	1	0	0	1	1	0	≥ 50 bp	12	12	10	9	14	16	14	11
SIM7	<50 bp	0	2	1	1	1	1	1	0	≥ 50 bp	10	9	8	7	12	10	11	8
SIM8	<50 bp	0	0	0	0	0	0	0	0	≥ 50 bp	14	8	8	5	8	13	6	8
SIM9	<50 bp	0	1	1	0	0	2	1	0	≥ 50 bp	8	11	12	13	17	21	14	12
SIM10	<50 bp	0	1	2	2	2	2	2	0	≥ 50 bp	14	13	10	10	11	16	12	5
SIM11	<50 bp	0	3	2	1	1	2	2	0	≥ 50 bp	10	14	12	15	19	13	13	8
SIM12	<50 bp	0	1	0	0	0	0	0	0	≥ 50 bp	12	11	11	9	12	14	12	6
SIM13	<50 bp	0	2	2	1	2	2	2	0	≥ 50 bp	13	10	9	10	14	14	9	14
SIM14	<50 bp	1	4	2	2	1	2	2	0	≥ 50 bp	8	12	11	13	16	15	10	8
SIM15	<50 bp	1	2	1	1	1	2	1	0	≥ 50 bp	5	14	13	9	13	16	12	5

SIM16	<50 bp	0	3	2	1	2	2	2	0	≥ 50 bp	8	13	9	7	10	14	13	8
SIM17	<50 bp	1	1	0	0	0	0	0	0	≥ 50 bp	18	8	6	7	6	13	12	9
SIM18	<50 bp	0	1	0	0	0	1	0	0	≥ 50 bp	12	19	14	15	21	20	17	10
SIM19	<50 bp	0	1	2	1	2	3	2	0	≥ 50 bp	7	10	9	10	13	11	16	13
SIM20	<50 bp	1	1	1	1	2	1	1	0	≥ 50 bp	14	10	9	9	11	13	11	12
Average		0	2	1	1	1	1	1	0		10	11	9	9	12	14	12	9
Error rate		0.03%	0.18%	0.12%	0.09%	0.09%	0.15%	0.14%	0.00%		0.60%	0.84%	0.73%	0.74%	0.96%	1.20%	1.00%	2.31%
**Missing rate**																		
SIM0	<50 bp	82	66	68	68	61	71	69	69	≥ 50 bp	10	1	0	0	1	3	2	1
SIM1	<50 bp	22	67	67	66	58	75	68	62	≥ 50 bp	6	8	6	11	9	9	9	41<br>3
SIM2	<50 bp	14	54	58	58	53	55	64	70	≥ 50 bp	4	10	8	13	11	9	9	1
SIM3	<50 bp	21	56	58	61	55	69	63	57	≥ 50 bp	7	8	5	8	10	9	11	31
SIM4	<50 bp	36	64	65	72	65	74	67	63	≥ 50 bp	7	10	8	13	12	7	10	33
SIM5	<50 bp	21	65	65	70	66	83	68	65	≥ 50 bp	5	11	8	16	12	12	8	32
SIM6	<50 bp	22	54	58	58	55	64	66	64	≥ 50 bp	8	5	5	7	8	10	9	28
SIM7	<50 bp	25	53	60	61	57	65	62	60	≥ 50 bp	11	10	8	16	13	15	12	27
SIM8	<50 bp	24	56	60	61	62	69	69	61	≥ 50 bp	8	11	7	7	10	8	7	27
SIM9	<50 bp	28	55	54	65	57	77	73	72	≥ 50 bp	8	10	9	7	13	13	9	29
SIM10	<50 bp	18	68	68	67	62	71	76	66	≥ 50 bp	9	9	7	12	9	9	7	32
SIM11	<50 bp	17	53	64	56	54	72	70	61	≥ 50 bp	6	7	8	14	12	9	10	38
SIM12	<50 bp	26	58	64	65	61	70	64	60	≥ 50 bp	5	4	4	9	9	9	4	34
SIM13	<50 bp	24	59	60	60	55	74	64	69	≥ 50 bp	5	6	3	7	4	8	4	27
SIM14	<50 bp	19	57	60	65	58	69	63	56	≥ 50 bp	6	7	5	11	9	9	10	31
SIM15	<50 bp	20	59	64	63	54	67	71	62	≥ 50 bp	6	4	4	7	7	6	11	33
SIM16	<50 bp	22	62	69	71	66	63	66	64	≥ 50 bp	6	12	9	12	14	13	11	25
SIM17	<50 bp	26	60	54	65	60	68	67	67	≥ 50 bp	5	7	4	9	10	8	8	32
SIM18	<50 bp	23	46	44	54	53	67	67	65	≥ 50 bp	6	7	6	8	12	10	10	31
SIM19	<50 bp	26	64	59	68	61	66	69	62	≥ 50 bp	6	9	5	9	9	11	10	30
SIM20	<50 bp	23	62	61	67	57	71	70	57	≥ 50 bp	8	9	8	11	13	10	11	34

**Average**		26	59	61	64	59	70	67	63		7	8	6	10	10	9	9	30
**Missing rate**		2.03%	6.59%	6.77%	7.33%	6.46%	8.29%	8.41%	30.49%		0.40%	0.62%	0.48%	0.82%	0.79%	0.82%	0.75%	7.92%

Accuracy, error, and missing rate for benchmarking analysis for deletions and insertions using *Solanum* phylogeny-based data.

		Insertions									Deletions							
		**SL4**	**M82**	**ZY65**	**ZY56**	**ZY57**	**ZY60**	**ZY61**	**ZY59**		**SL4**	**M82**	**ZY65**	**ZY56**	**ZY57**	**ZY60**	**ZY61**	**ZY59**
**# of retrieved calls**		**3,356**	**3,245**	**3,149**	**3,036**	**2,906**	**1,695**	**1,340**	**1,106**		**2,978**	**2,815**	**2,774**	**2,755**	**2,566**	**1,748**	**1,406**	**1,034**
**Accuracy**																		
SIM0	≥50 bp	3,351	3,241	3,144	3,031	2,900	1,691	1,338	1,104	≥ 50 bp	2,978	2,815	2,774	2,755	2,566	1,747	1,405	1,032
SIM1	≥50 bp	3,353	3,240	3,145	3,020	2,883	1,630	1,249	1,060	≥ 50 bp	2,977	2,808	2,770	2,735	2,555	1,716	1,355	1,000
SIM2	≥50 bp	3,351	3,237	3,143	3,017	2,887	1,629	1,269	1,042	≥ 50 bp	2,977	2,808	2,771	2,740	2,554	1,721	1,357	1,001
SIM3	≥50 bp	3,354	3,240	3,147	3,021	2,880	1,630	1,272	1,058	≥ 50 bp	2,977	2,807	2,773	2,738	2,557	1,716	1,365	1,000
SIM4	≥50 bp	3,350	3,236	3,141	3,021	2,885	1,631	1,279	1,051	≥ 50 bp	2,977	2,809	2,772	2,738	2,559	1,715	1,348	1,003
SIM5	≥50 bp	3,352	3,237	3,145	3,022	2,884	1,633	1,269	1,054	≥ 50 bp	2,978	2,810	2,773	2,739	2,557	1,713	1,365	1,007
SIM6	≥50 bp	3,346	3,231	3,140	3,014	2,878	1,626	1,266	1,047	≥ 50 bp	2,978	2,813	2,771	2,734	2,556	1,710	1,358	1,006
SIM7	≥50 bp	3,351	3,234	3,144	3,020	2,885	1,624	1,269	1,045	≥ 50 bp	2,977	2,807	2,771	2,741	2,556	1,718	1,352	993
SIM8	≥50 bp	3,353	3,239	3,146	3,023	2,890	1,629	1,271	1,052	≥ 50 bp	2,977	2,808	2,772	2,732	2,555	1,713	1,359	1,003
SIM9	≥50 bp	3,352	3,237	3,144	3,021	2,886	1,628	1,262	1,048	≥ 50 bp	2,977	2,810	2,769	2,734	2,555	1,714	1,362	1,004
SIM10	≥50 bp	3,349	3,238	3,142	3,019	2,878	1,621	1,263	1,042	≥ 50 bp	2,977	2,810	2,770	2,741	2,558	1,717	1,360	1,005
SIM11	≥50 bp	3,353	3,239	3,146	3,020	2,885	1,634	1,269	1,047	≥ 50 bp	2,978	2,808	2,771	2,736	2,558	1,714	1,359	1,002
SIM12	≥50 bp	3,350	3,238	3,144	3,024	2,886	1,637	1,263	1,048	≥ 50 bp	2,978	2,807	2,772	2,739	2,554	1,721	1,356	1,012
SIM13	≥50 bp	3,350	3,238	3,143	3,010	2,883	1,624	1,266	1,053	≥ 50 bp	2,978	2,810	2,771	2,740	2,552	1,718	1,363	1,007
SIM14	≥50 bp	3,352	3,240	3,145	3,021	2,886	1,641	1,266	1,050	≥ 50 bp	2,977	2,806	2,772	2,741	2,554	1,715	1,363	1,003
SIM15	≥50 bp	3,351	3,236	3,144	3,017	2,883	1,633	1,269	1,054	≥ 50 bp	2,977	2,811	2,771	2,733	2,555	1,717	1,357	1,010
SIM16	≥50 bp	3,351	3,237	3,143	3,012	2,881	1,626	1,262	1,051	≥ 50 bp	2,977	2,807	2,768	2,736	2,551	1,714	1,365	1,007
SIM17	≥50 bp	3,348	3,234	3,140	3,018	2,883	1,622	1,272	1,045	≥ 50 bp	2,977	2,808	2,771	2,738	2,552	1,719	1,363	1,002
SIM18	≥50 bp	3,350	3,236	3,144	3,014	2,885	1,622	1,270	1,049	≥ 50 bp	2,977	2,808	2,771	2,737	2,555	1,708	1,359	1,008
SIM19	≥50 bp	3,351	3,234	3,142	3,012	2,882	1,623	1,264	1,043	≥ 50 bp	2,978	2,810	2,770	2,741	2,557	1,716	1,363	1,008
SIM20	≥50 bp	3,349	3,234	3,142	3,014	2,880	1,622	1,253	1,052	≥ 50 bp	2,977	2,810	2,772	2,742	2,557	1,712	1,361	998
**Average**		3,351	3,237	3,144	3,019	2,884	1,631	1,270	1,052		2,977	2,809	2,771	2,739	2,556	1,717	1,362	1,005
**Accuracy**		99.85%	99.75%	99.83%	99.43%	99.25%	96.24%	94.74%	95.13%		99.98%	99.79%	99.90%	99.40%	99.60%	98.22%	96.85%	97.22%

**Error rate**																		
**SIM0**	≥50 bp	0	0	0	0	1	0	0	0	≥ 50 bp	0	0	0	0	0	0	0	0
**SIM1**	≥50 bp	0	0	0	0	3	1	6	0	≥ 50 bp	1	1	1	6	1	2	3	6
**SIM2**	≥50 bp	0	0	0	2	2	0	3	1	≥ 50 bp	1	2	1	6	1	2	5	4
**SIM3**	≥50 bp	0	0	0	0	2	0	6	0	≥ 50 bp	1	2	1	8	1	1	3	6
**SIM4**	≥50 bp	0	0	0	1	4	1	2	1	≥ 50 bp	1	1	1	8	1	1	3	3
**SIM5**	≥50 bp	0	0	0	0	3	0	6	0	≥ 50 bp	0	0	0	5	0	1	2	3
**SIM6**	≥50 bp	0	0	0	2	2	0	2	0	≥ 50 bp	0	0	0	6	0	0	4	2
**SIM7**	≥50 bp	0	0	0	2	3	1	7	1	≥ 50 bp	1	2	1	5	1	2	6	5
**SIM8**	≥50 bp	0	0	0	0	2	1	2	0	≥ 50 bp	1	1	1	8	1	1	3	4
**SIM9**	≥50 bp	0	0	0	0	3	1	2	0	≥ 50 bp	1	1	1	6	1	2	2	5
**SIM10**	≥50 bp	0	0	0	1	2	0	6	0	≥ 50 bp	1	1	1	5	1	1	2	5
**SIM11**	≥50 bp	0	0	0	1	3	0	4	1	≥ 50 bp	0	1	0	7	0	1	2	3
**SIM12**	≥50 bp	0	0	0	0	1	0	2	0	≥ 50 bp	0	1	0	6	0	1	2	2
**SIM13**	≥50 bp	0	0	0	1	4	1	3	0	≥ 50 bp	0	1	0	3	0	0	2	2
**SIM14**	≥50 bp	0	0	0	1	2	2	6	0	≥ 50 bp	1	1	1	5	1	2	4	3
**SIM15**	≥50 bp	0	0	0	0	3	1	3	0	≥ 50 bp	1	1	1	10	1	2	2	4
**SIM16**	≥50 bp	0	0	0	1	1	1	3	0	≥ 50 bp	1	2	1	8	1	2	3	4
**SIM17**	≥50 bp	0	0	0	1	3	2	2	0	≥ 50 bp	1	1	1	8	1	1	3	3
**SIM18**	≥50 bp	0	0	0	1	2	2	2	1	≥ 50 bp	1	2	1	8	1	2	4	2
**SIM19**	≥50 bp	0	0	0	1	2	0	8	0	≥ 50 bp	0	1	0	2	0	1	0	4
**SIM20**	≥50 bp	0	0	0	1	1	1	3	0	≥ 50 bp	1	1	1	9	1	2	3	6
**Average**		0	0	0	1	2	1	4	0		1	1	1	6	1	1	3	4
**Error rate**		0.00%	0.00%	0.00%	0.03%	0.08%	0.04%	0.28%	0.02%		0.02%	0.04%	0.02%	0.22%	0.03%	0.07%	0.20%	0.35%
**Missing rate**																		
**SIM0**	≥50 bp	5	4	5	5	5	4	2	2	≥ 50 bp	0	0	0	0	0	1	1	2
**SIM1**	≥50 bp	3	5	4	16	20	64	85	46	≥ 50 bp	0	6	3	14	10	30	48	28
**SIM2**	≥50 bp	5	8	6	17	17	66	68	63	≥ 50 bp	0	5	2	9	11	25	44	29
**SIM3**	≥50 bp	2	5	2	15	24	65	62	48	≥ 50 bp	0	6	0	9	8	31	38	28
**SIM4**	≥50 bp	6	9	8	14	17	63	59	54	≥ 50 bp	0	5	1	9	6	32	55	28

**SIM5**	≥50 bp	4	8	4	14	19	62	65	52	≥ 50 bp	0	5	1	11	9	34	39	24
**SIM6**	≥50 bp	10	14	9	20	26	69	72	59	≥ 50 bp	0	2	3	15	10	38	44	26
**SIM7**	≥50 bp	5	11	5	14	18	70	64	60	≥ 50 bp	0	6	2	9	9	28	48	36
**SIM8**	≥50 bp	3	6	3	13	14	65	67	54	≥ 50 bp	0	6	1	15	10	34	44	27
**SIM9**	≥50 bp	4	8	5	15	17	66	76	58	≥ 50 bp	0	4	4	15	10	32	42	25
**SIM10**	≥50 bp	7	7	7	16	26	74	71	64	≥ 50 bp	0	4	3	9	7	30	44	24
**SIM11**	≥50 bp	3	6	3	15	18	61	67	58	≥ 50 bp	0	6	3	12	8	33	45	29
**SIM12**	≥50 bp	6	7	5	12	19	58	75	58	≥ 50 bp	0	7	2	10	12	26	48	20
**SIM13**	≥50 bp	6	7	6	25	19	70	71	53	≥ 50 bp	0	4	3	12	14	30	41	25
**SIM14**	≥50 bp	4	5	4	14	18	52	68	56	≥ 50 bp	0	8	1	9	11	31	39	28
**SIM15**	≥50 bp	5	9	5	19	20	61	68	52	≥ 50 bp	0	3	2	12	10	29	47	20
**SIM16**	≥50 bp	5	8	6	23	24	68	75	55	≥ 50 bp	0	6	5	11	14	32	38	23
**SIM17**	≥50 bp	8	11	9	17	20	71	66	61	≥ 50 bp	0	6	2	9	13	28	40	29
**SIM18**	≥50 bp	6	9	5	21	19	71	68	56	≥ 50 bp	0	5	2	10	10	38	43	24
**SIM19**	≥50 bp	5	11	7	23	22	72	68	63	≥ 50 bp	0	4	4	12	9	31	43	22
**SIM20**	≥50 bp	7	11	7	21	25	72	84	54	≥ 50 bp	0	4	1	4	8	34	42	30
**Average**		5	8	5	17	19	63	67	54		0	5	2	10	9	30	42	25
**Missing rate**		0.15%	0.25%	0.17%	0.55%	0.67%	3.72%	4.98%	4.85%		0.00%	0.17%	0.08%	0.37%	0.37%	1.71%	2.96%	2.43%

Supplementary Table 11: Benchmark SV detection in maize using SVGAP and paftools.

**Methods**	**# of simulated SVs**	**# of Predicted SVs**	**Recall**	**Accuracy**	**F1 score**
Simulated 2000 SVs (deletions and insertions) on chr10 without additional mutations at the flanking region of breakpoints					
minimap2_default	2,000	2,053	1,837/2,000 (91.85%)	1,837/2,053 (89.5%)	**90.65%**
minimap2_asm5	2,000	2,601	1,740/2,000 (87%)	1,740/2,601 (66.9%)	75.64%
minimap2_asm10	2,000	2,343	1,894/2,000 (94.7%)	1,894/2,343 (80.8%)	87.22%
minimap2_asm20	2,000	2,245	1,906/2,000 (95.3%)	1,906/2,245 (84.9%)	89.80%
paftools_default	2,000	1,581	1,488/2,000 (74.4%)	1,488/1,581 (94.1%)	83.11%
paftools_asm5	2,000	1,110	980/2,000 (49%)	980/1,110 (88.3%)	63.02%
paftools_asm10	2,000	1,683	1,553/2,000 (77.65%)	1,553/1,683 (92.3%)	84.33%
paftools_asm20	2,000	1,603	1,525/2,000 (76.25%)	1,525/1,603 (95.1%)	84.65%
Simulated 3000 SVs (deletions and insertions) on chr10 without additional mutations at the flanking region of breakpoints					
minimap2_default	3,000	2,979	2,944/3,000 (98.1%)	2,944/2,979 (98.8%)	**98.48%**
minimap2_asm5	3,000	2,963	2,931/3,000 (97.7%)	2,931/2,963 (98.9%)	98.31%
minimap2_asm10	3,000	2,963	2,933/3,000 (97.8%)	2,933/2,963 (99.0%)	98.37%
minimap2_asm20	3,000	2,969	2,933/3,000 (97.8%)	2,933/2,969 (98.8%)	98.27%
paftools_default	3,000	4,584	1,896/3,000 (63.2%)	1,896/4,584 (41.4%)	50.00%
paftools_asm5	3,000	2,058	1,907/3,000 (63.6%)	1,907/2,058 (92.7%)	75.41%
paftools_asm10	3,000	2,423	1,907/3,000 (63.6%)	1,907/2,423 (78.7%)	70.33%
paftools_asm20	3,000	2,178	1,887/3,000 (62.9%)	1,887/2,178 (86.6%)	72.89%

Recall was determined based on an 80% reciprocal overlap cutoff.

Supplementary Table 12: SV detected between 25 NAM maize lines to the reference B73 using SVGAP.

Pairwise comparison	DEL	INS	DUP	INV	TRL
**B73 vs. Ab10**	1,316	3,931	54	28	58
**B73 vs. B97**	28,955	34,928	427	80	379
**B73 vs. CML103**	32,127	36,620	433	87	376
**B73 vs. CML228**	33,229	38,509	548	93	349
**B73 vs. CML247**	33,688	38,876	511	105	365
**B73 vs. CML277**	33,391	37,726	479	91	359
**B73 vs. CML322**	32,660	38,546	466	125	389
**B73 vs. CML333**	32,039	38,515	484	114	417
**B73 vs. CML52**	31,792	39,508	576	102	397
**B73 vs. CML69**	33,093	38,463	461	111	377
**B73 vs. HP301**	31,358	34,879	461	91	359
**B73 vs. Il14H**	32,426	36,064	469	87	388
**B73 vs. Ki11**	33,394	41,436	498	147	438
**B73 vs. Ki3**	33,203	37,768	479	102	375
**B73 vs. Ky21**	29,168	32,941	466	87	335
**B73 vs. M162W**	31,780	36,891	440	83	346
**B73 vs. M37W**	31,663	36,690	434	96	344
**B73 vs. Mo18W**	32,419	38,794	492	115	393
**B73 vs. Ms71**	30,084	35,386	441	81	361
**B73 vs. NC350**	33,096	42,040	504	135	473
**B73 vs. NC358**	32,360	38,234	499	99	353
**B73 vs. Oh43**	29,835	34,063	457	111	356
**B73 vs. Oh7b**	28,266	32,372	431	78	304
**B73 vs. P39**	32,819	36,596	466	119	405
**B73 vs. Tx303**	31,745	37,879	485	115	375
**B73 vs. Tzi8**	32,645	38,435	558	93	379
**Total**	798,551	936,090	12,019	2,575	9,450
**Non-redundant**	192,455	312,188	6,612	2,084	-

Supplementary Table 13: Runtime and memory requirements for each step in SVGAP applied to 49 rice genomes.

Pipeline steps	User time (Hour)	System time (Hour)	Elapsed (wall clock) time (Hour)	Maximum memory (Gb)	Threads
Alignment per sample (minimap2)	1.28	0.02	0.16	71.29	40
1_Convert2Axt	0.40	0.05	0.02	0.45	40
2_ChainNetSyn	0.34	0.03	0.37	0.78	-
3_SynNetFilter	0.16	0.03	0.19	0.78	-
4_PairwiseSV	136.14	0.12	4.41	1.19	40
5_Combined	3.11	0.05	2.47	5.51	-
6_DELGenoptyping	430.35	244.63	63.85	15.07	12
7_INSGenotyping	279.25	384.91	57.18	15.16	12
8_InDelGenotyping_del	10.04	0.47	1.39	15.68	12
8_InDelGenotyping_ins	9.53	0.44	1.26	15.07	12
9_SNPGenotyping	11.22	0.52	1.71	15.16	12

Supplementary Figures

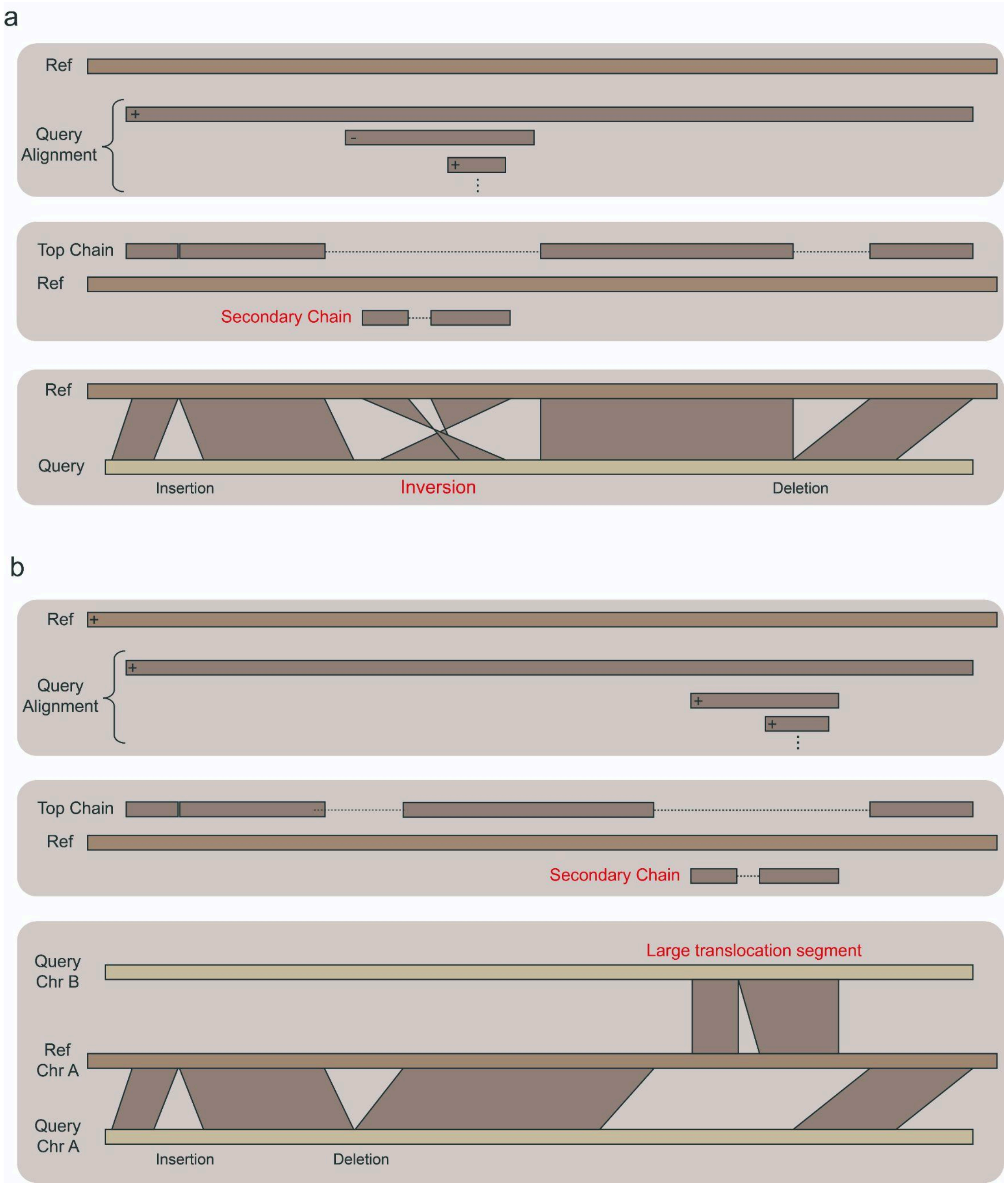

Supplementary Figure 1: SVGAP facilitates structural variant (SV) identification in inversion and large translocation segments. In most cases, SVs were detected from the sequence alignments in the top chain. However, some regions exhibited large inversions (**a**) or translocations (**b**), which are more likely to appear as lower (i.e., secondary) chains. These regions are rearranged genomic areas that remain syntenic and orthologous, indicating that the SVs within them should be identified. SVGAP is capable of identifying SVs in these regions and constructing a comprehensive SV dataset.

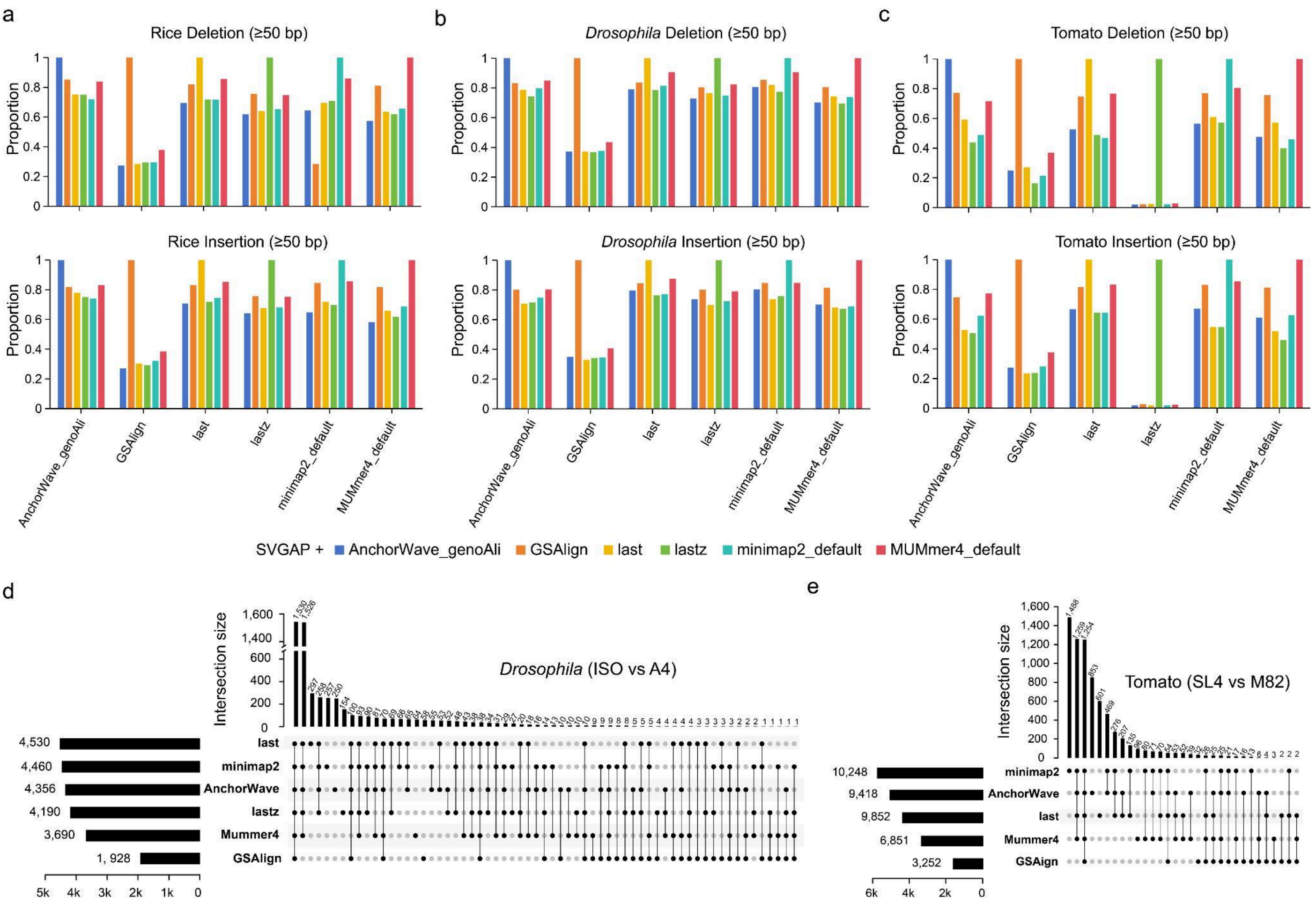

Supplementary Figure 2: Comparison of SVGAP with different aligners for identifying SVs between real genomes in rice, *Drosophila*, and tomato. a. Pairwise overlap of SVs (deletions and insertions) identified by SVGAP using alignment results from different aligners between rice genome assemblies: MH63 and ZS97. **b**. Pairwise overlap of SVs identified by SVGAP using alignment results from different aligners between two *Drosophila* genome assemblies: iso-1 and A4. **c**. Pairwise overlap of SVs identified by SVGAP using alignment results from different aligners between two tomato genome assemblies: SL4 and M82. **d**. An upset plot showing the analysis of shared calls between different aligners on two Drosophila data. **e**. An upset plot showing the analysis of shared calls between different aligners on tomato data. The exact numbers are available in Supplementary Table 4.

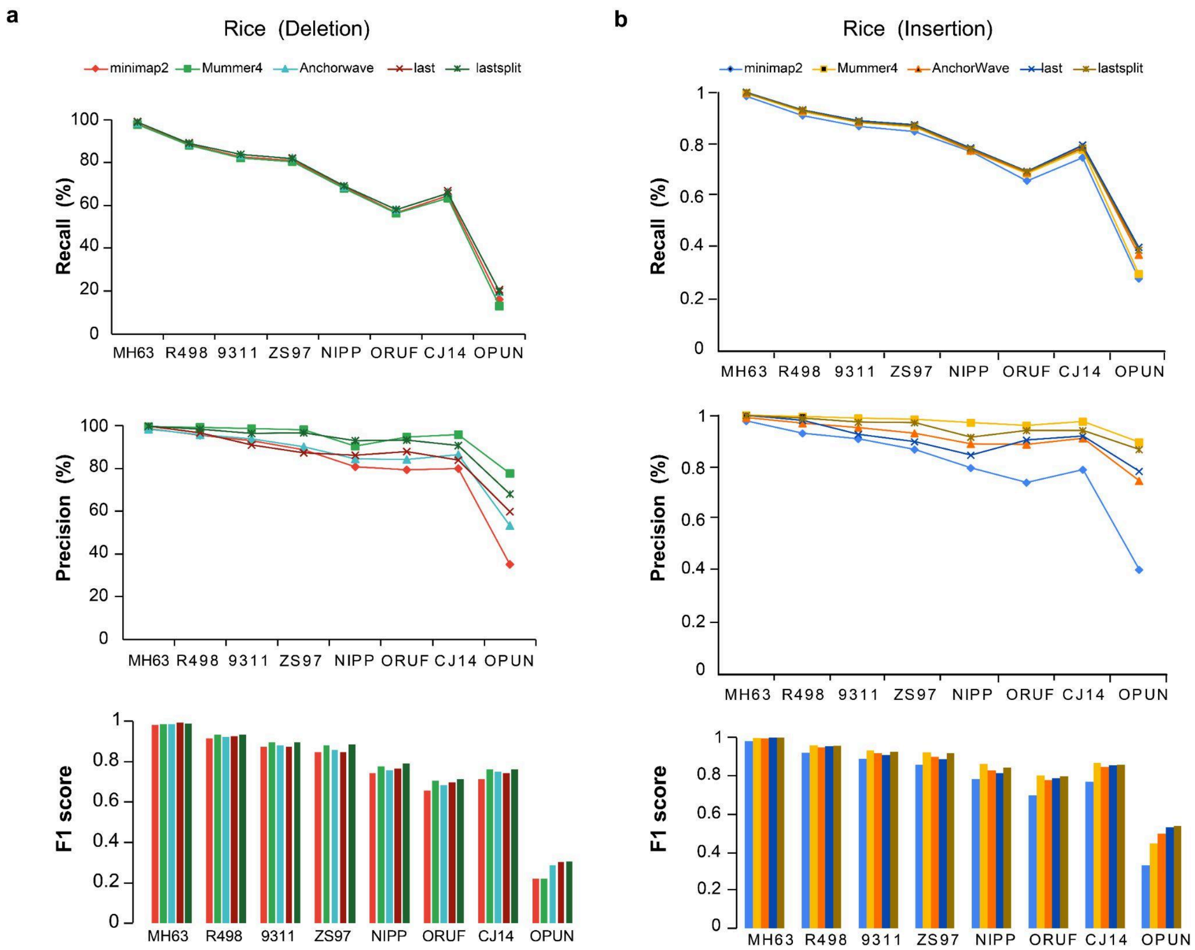

minimap2	0.9826	0.9170	0.8755	0.8462	0.7420	0.6593	0.7147	0.2201
Mummer4	0.9854	0.9329	0.8962	0.8833	0.7754	0.7065	0.7629	0.2202
Anchorwave	0.9848	0.9219	0.8826	0.8588	0.7581	0.6855	0.7500	0.2866
last	0.9938	0.9270	0.8727	0.8465	0.7660	0.6977	0.7437	0.3051
last + split	0.9915	0.9333	0.8959	0.8851	0.7925	0.7142	0.7621	0.3073

minimap2	0.9807	0.9194	0.8878	0.8570	0.7834	0.6957	0.7675	0.3299
Mummer4	0.9970	0.9579	0.9324	0.9210	0.8614	0.8001	0.8655	0.4448
AnchorWave	0.9942	0.9479	0.9166	0.8995	0.8281	0.7758	0.8451	0.4963
last	0.9987	0.9549	0.9074	0.8865	0.8134	0.7859	0.8531	0.5285
last+split	0.9982	0.9571	0.9266	0.9172	0.8415	0.7970	0.8559	0.5366

Supplementary Figure 3: Benchmarking SVGAP with different aligners for SV calling using *Oryza* phylogeny-based simulation data. a. Recall, precision, and F1 score for deletions. **b**. Recall, precision, and F1 score for insertions. The exact numbers are available in Supplementary Table 6.

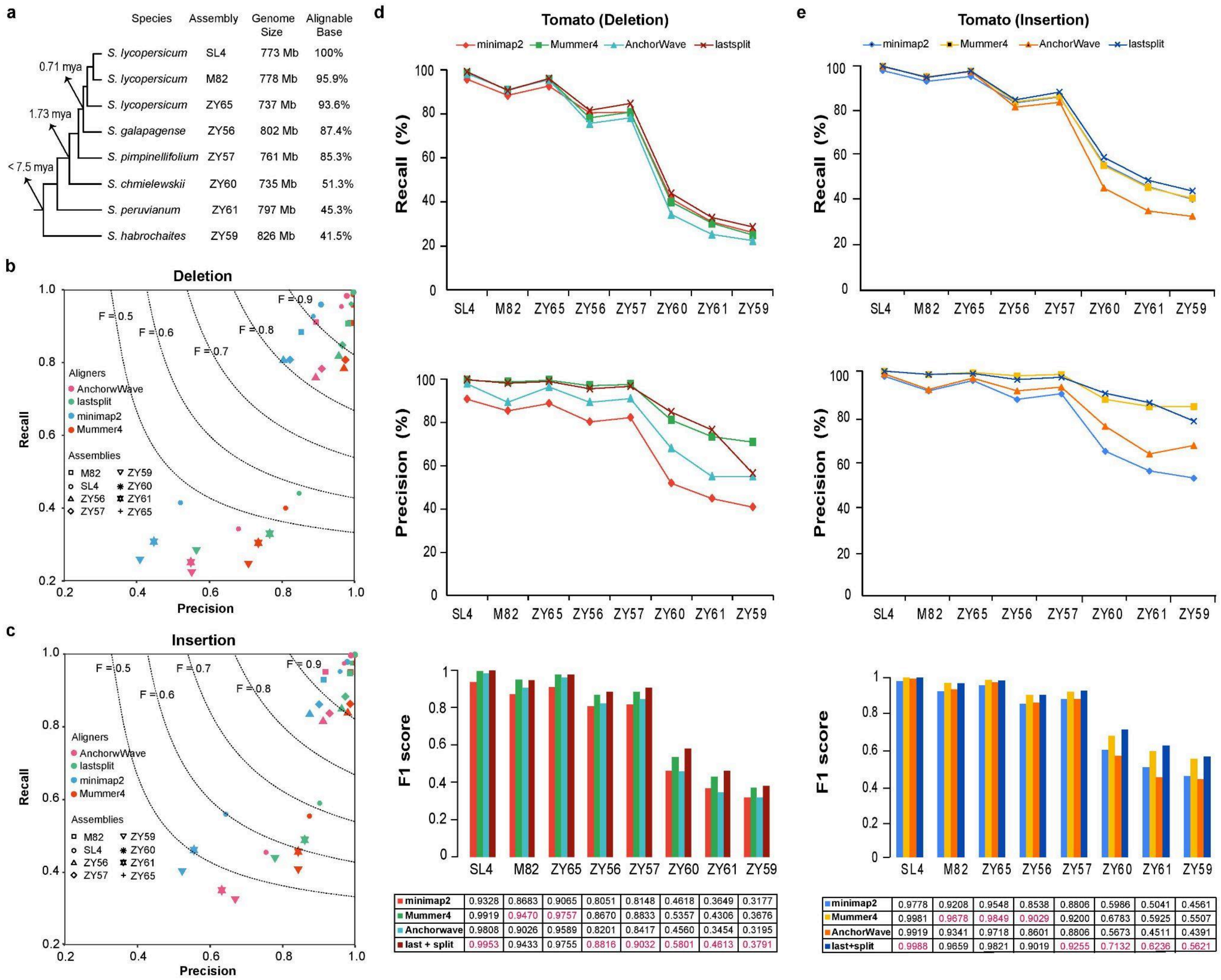

Supplementary Figure 4: Benchmarking SVGAP with different aligners for SV calling using *Solanum* phylogeny-based simulation data. a. The *Solanum* genomes, phylogenetic relationship and sequence divergence used for benchmark analysis. **b.** Recall, precision, and F1 score for deletions. **c**. Recall, precision, and F1 score for insertions. **d** and **e** showing the trend of each metric changes as sequence divergence. The exact numbers are available in Supplementary Table 6.

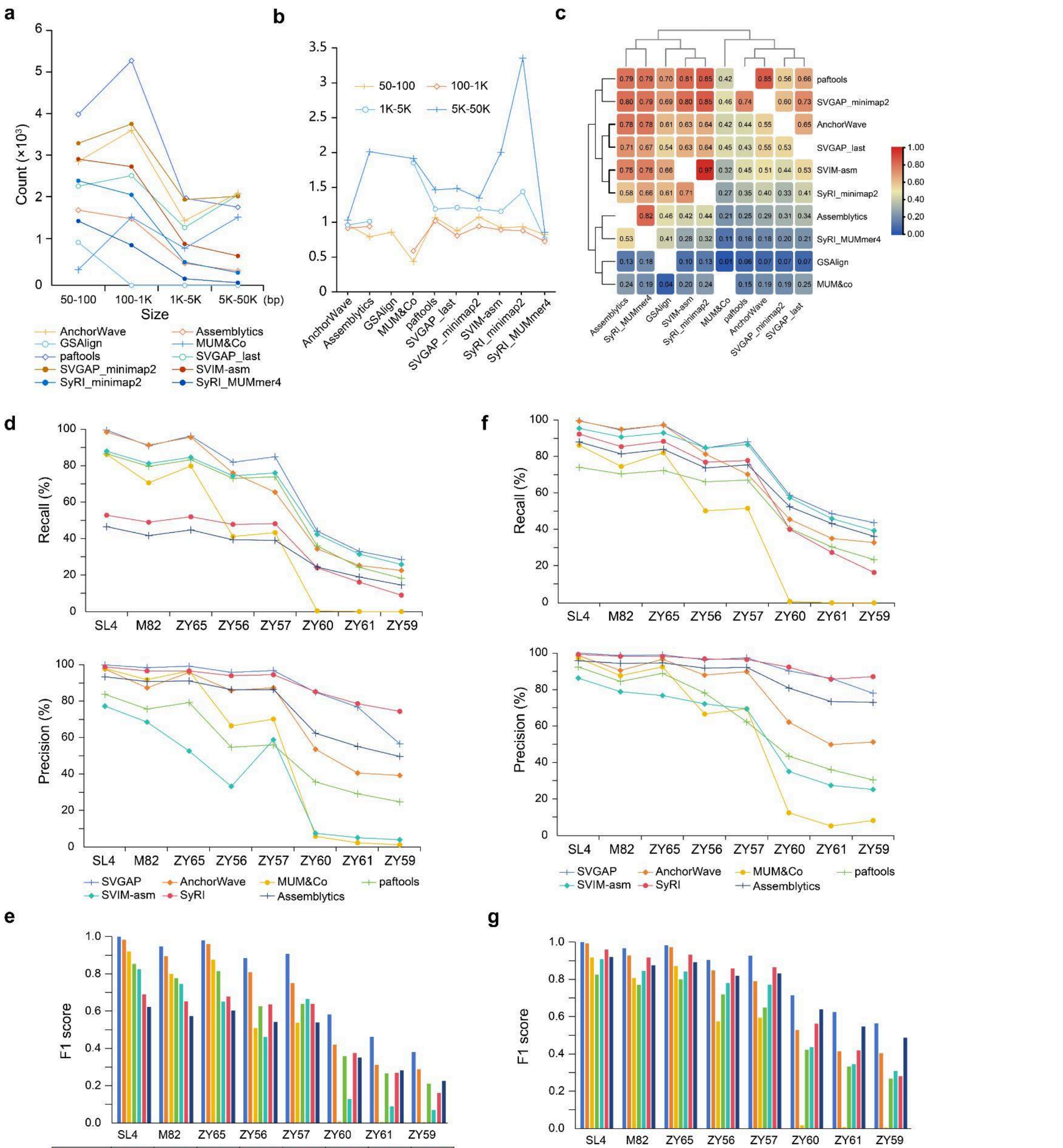

	SL4	M82	ZY65	ZY56	ZY57	ZY60	ZY61	ZY59
SVGAP	0.9953	0.9433	0.9755	0.8816	0.9032	0.5801	0.4613	0.3791
AnchorWave	0.9787	0.8911	0.9554	0.8048	0.7473	0.4185	0.3111	0.2862
MUM&Co	0.9158	0.7969	0.8729	0.5075	0.535	0.0074	0.0007	0.0002
paftools	0.8498	0.7748	0.8107	0.6247	0.6362	0.3579	0.2648	0.21
SVIM-asm	0.8213	0.7421	0.6479	0.4587	0.6624	0.1275	0.0882	0.0687
SyRI	0.6875	0.6495	0.6751	0.6329	0.6375	0.3747	0.2679	0.1605
Assemblytics	0.6207	0.5703	0.5995	0.5398	0.5377	0.3505	0.282	0.2249

	SL4	M82	ZY65	ZY56	ZY57	ZY60	ZY61	ZY59
SVGAP	0.9988	0.9659	0.9821	0.9019	0.9255	0.7132	0.6236	0.5621
AnchorWave	0.991	0.927	0.97	0.8458	0.7895	0.5272	0.4135	0.4021
MUM&Co	0.9148	0.8063	0.8706	0.5741	0.5936	0.0157	0.002	0.0011
paftools	0.8233	0.7692	0.7989	0.7174	0.647	0.4209	0.3317	0.2668
SVIM-asm	0.9067	0.8441	0.841	0.7801	0.7708	0.4362	0.3447	0.3082
SyRI	0.9573	0.9146	0.9309	0.8581	0.8625	0.5612	0.4182	0.2805
Assemblytics	0.918	0.8749	0.8908	0.8187	0.8306	0.6379	0.5461	0.4866

Supplementary Figure 5: Comparison of SVGAP with other methods using benchmark analysis based on simulated data from tomato and *Solanum* phylogeny. a. Number of SV calls reported by different methods across various length ranges when comparing the two tomato genomes, SL4 and M82. **b**. Ratio of deletions to insertions reported by different methods based on the two tomato genomes. **c**. Overlap of SVs in pairwise comparisons among different methods. **d**. Recall and precision for the benchmark analysis of deletions using simulated data from *Solanum* phylogeny. **e**. F1 score for deletions. **f**. Recall and precision for insertions. **g**. F1 score for insertions.

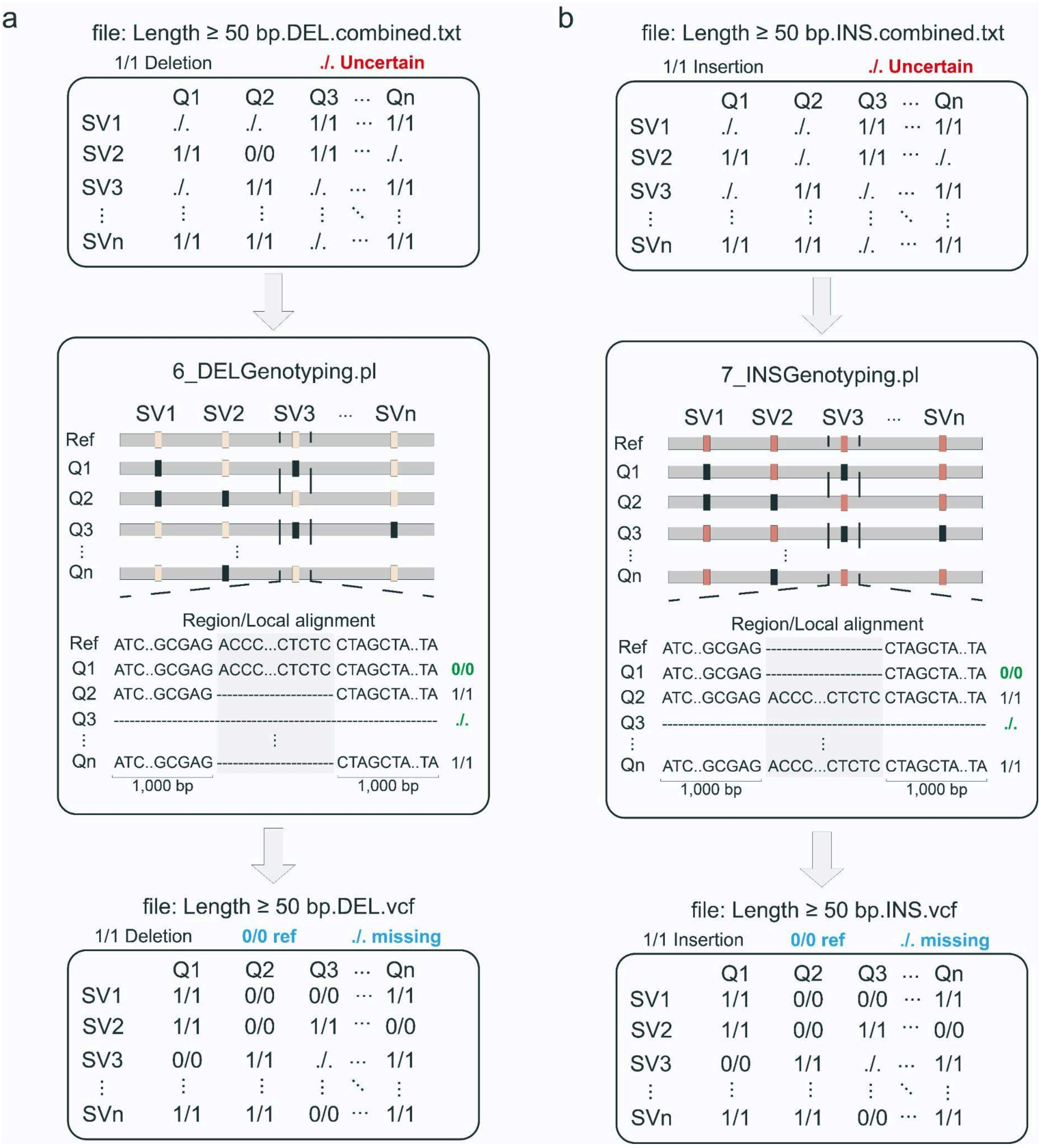

Supplementary Figure 6: The diagram illustrates the strategy of population genotyping for deletions and insertions. The genotyping step takes a pre-combined SV dataset as input and re-genotypes each SV call across all samples by extracting local alignments around the SV site from the corresponding pairwise single-coverage genome alignments. For each sample and SV, SVGAP reports three genotypes: 1/1 (alternative type), 0/0 (reference type), and ./. (missing or unable to determine).

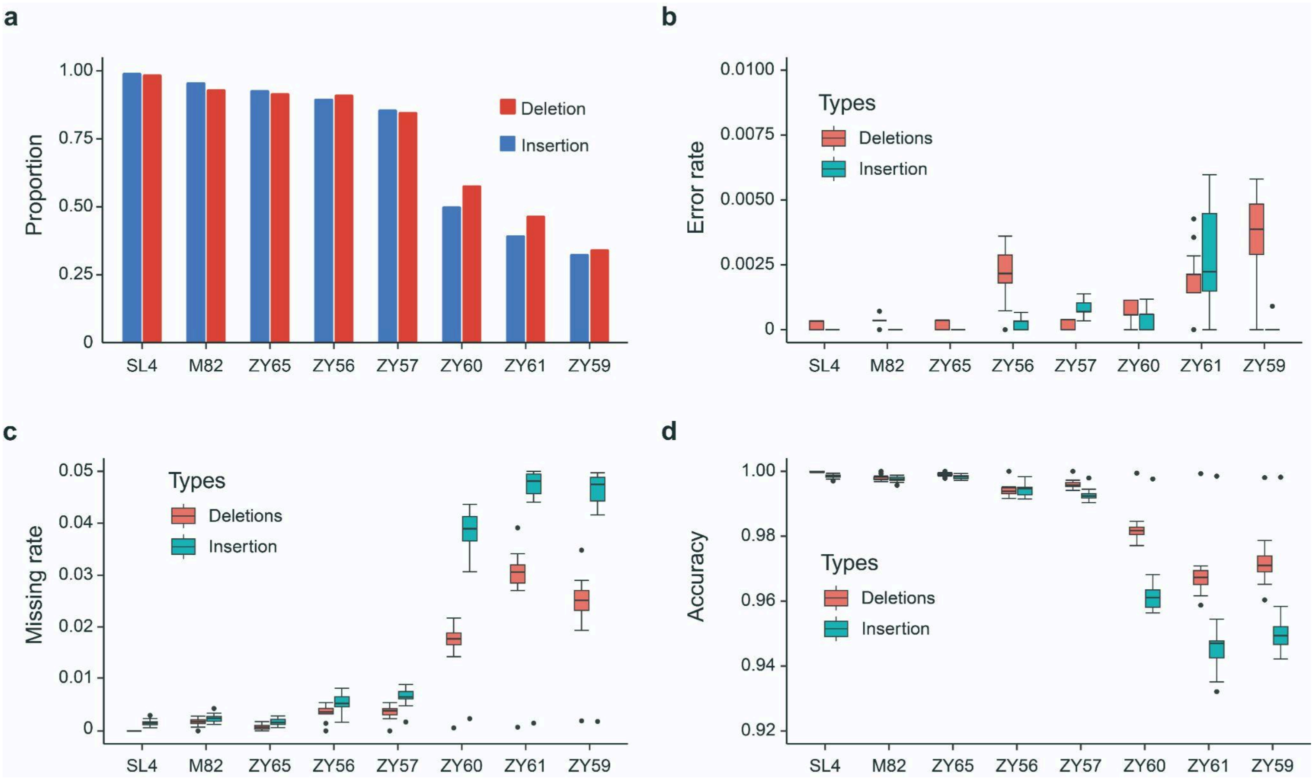

Supplementary Figure 7: Benchmark analysis of the merging and genotyping functions in SVGAP with tomato data. a, Proportion of retrieved simulated calls in the merging step by comparing the reference SL4 and eight other *Solanum* genomes of varying sequence divergence. **b**, Error rate of the genotyping step with population-scale simulating data based on *Solanum* phylogeny. **c**, The missing rate of the genotyping step across genomes of varying sequence divergence. **d**. The accuracy of the genotyping step across genomes of varying sequence divergence.

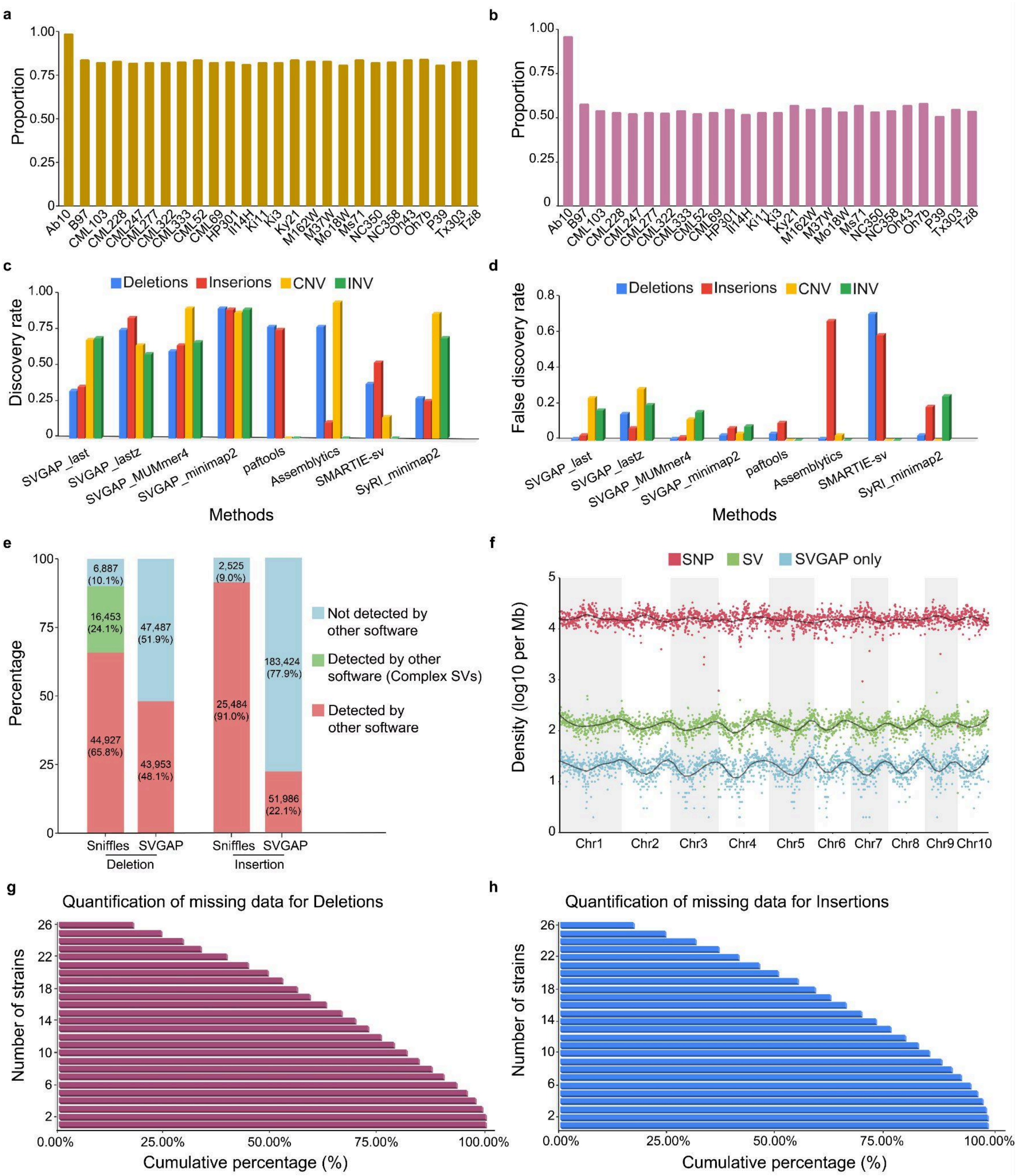

Supplementary Figure 8: Structural variant (SV) identification across 26 maize genomes. a. Proportion of genome regions alignable between B73 and other 26 maize genomes. **b.** Proportion of genome regions alignable in synteny between B73 and other 26 maize genomes. **c.** Recall (discovery rate) for benchmarking SV detection across different SV types in maize. **d.** False discovery rate (FDR) for benchmarking SV detection across SV types in maize. **e.** Comparison of SV calls between a previous study [10] using Sniffles and SVGAP calls from this study. **f.** Chromosome-level distribution of SVs and SNPs, highlighting SVs uniquely detected by SVGAP. **g.** Cumulative proportion of deletions identified across 26 maize genomes (based on chromosome 1 deletions). **h.** Cumulative proportion of insertions identified across 26 maize genomes (based on chromosome 2 insertions).

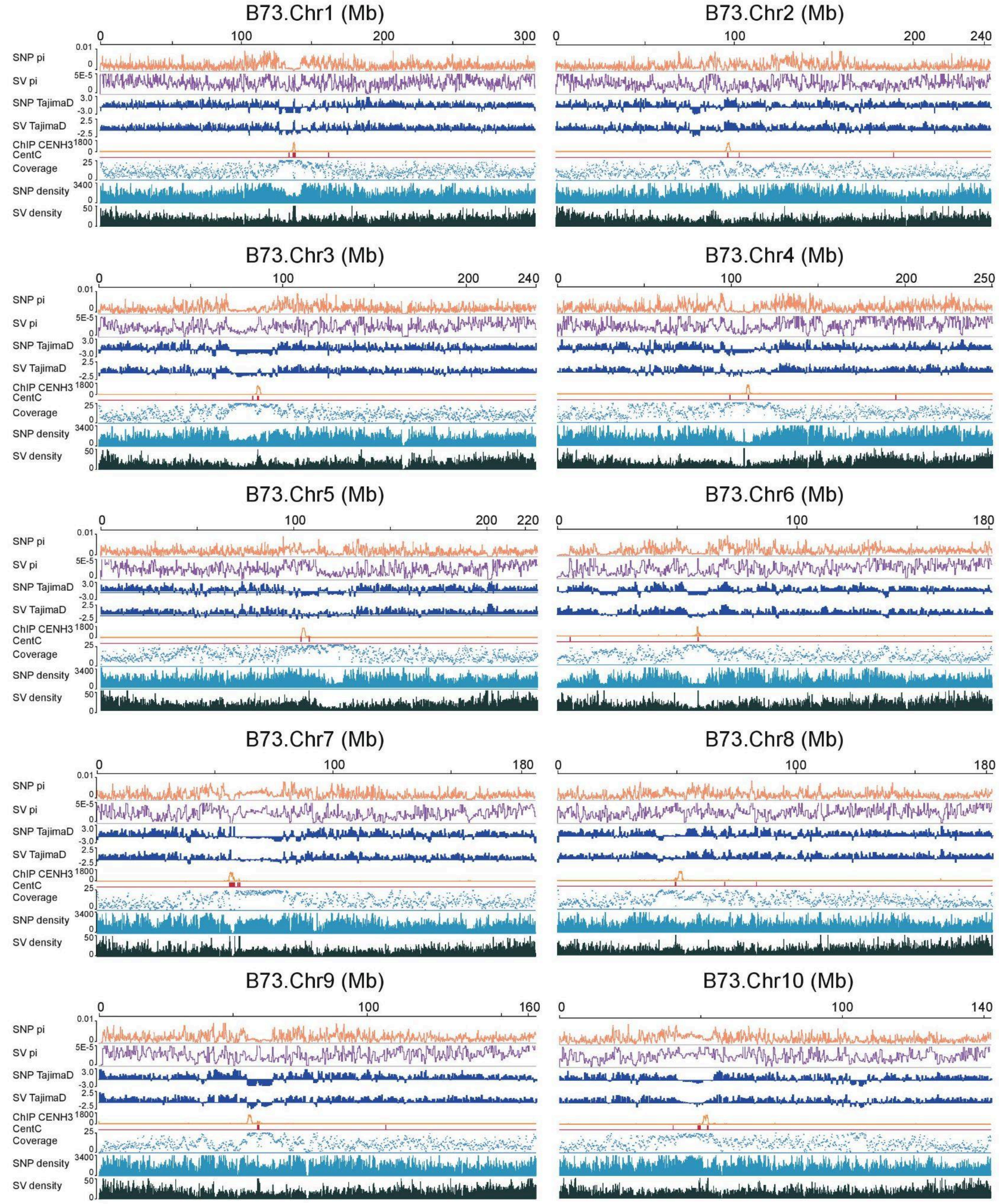

Supplementary Figure 9: Population properties of genetic variants identified by SVGAP across the maize genome. The average pairwise diversity (π), Tajima's D, as well as SNP and SV density were calculated in 100-kb non-overlapping windows along each chromosome. Coverage is represented by the number of genomes that can be syntenically aligned to the B73 reference, calculated for each 10-kb window. Notably, an elevated coverage was observed around the functional centromeric regions across most chromosomes, highlighting the structural and evolutionary conservation of these regions.

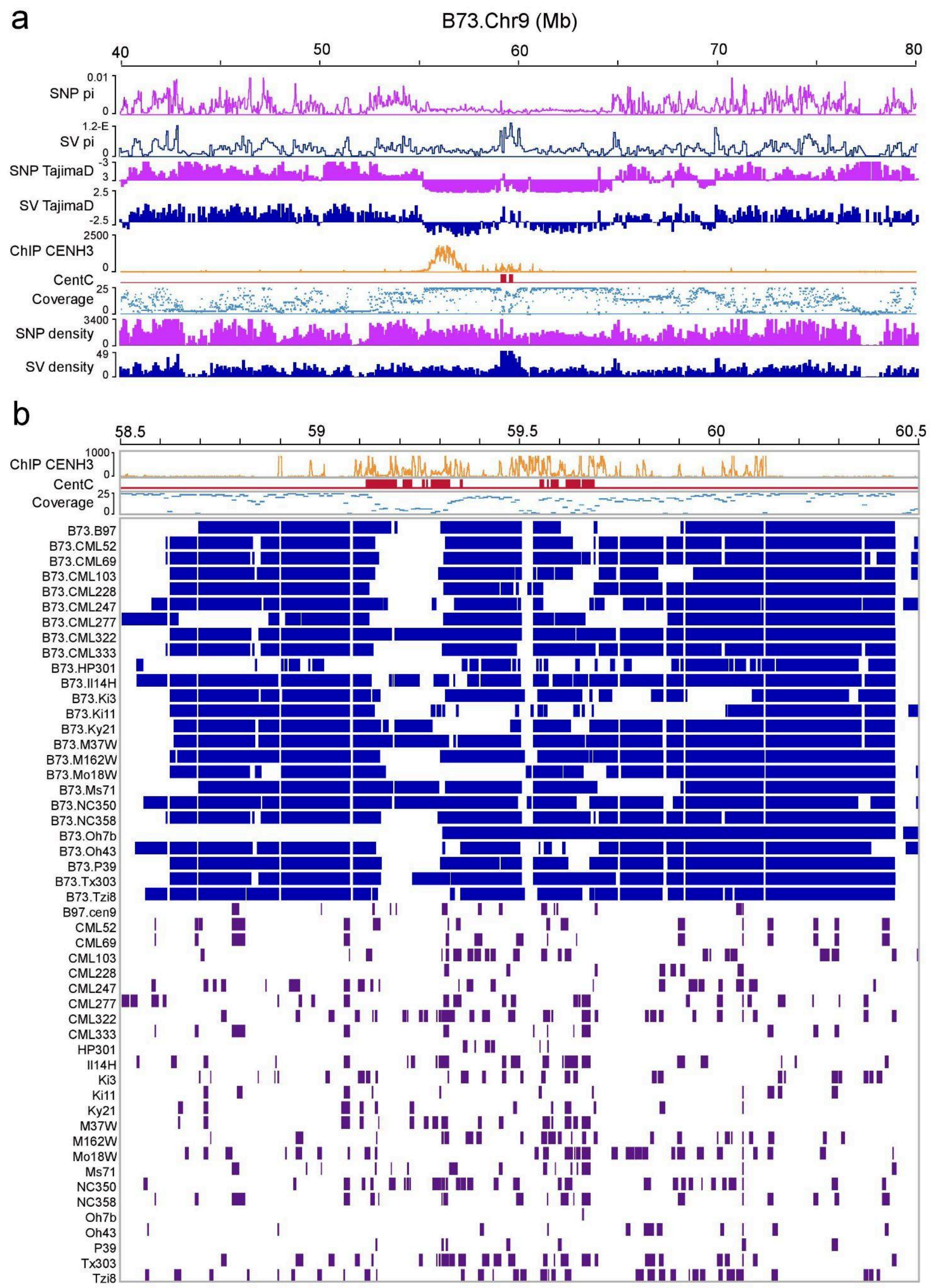

Supplementary Figure 10: Features of genetic variations and their population properties around the functional centromeric regions of chromosome 9. a. Panels from top to bottom include: (1) average pairwise diversity (π) for SNPs calculated in non-overlapping 100-bp windows along the chromosome; (2) π calculated for structural variants (SVs); (3) Tajima's D calculated with SNPs; (4) Tajima's D calculated with SVs; (5) CENH3 ChIP-seq peaks; (6) distribution of centromere-specific tandem repeats (CentC); (7) number of genomes aligned to the B73 genome in each 10-kb window; (8) SNP density; and (9) SV density. **b.** Functional centromeric regions are enriched with insertions of centromere-specific retrotransposable elements. Both sequence alignment coverage (blue) and the presence of transposable element (TE) insertions (purple) are shown for 26 maize genomes relative to the B73 reference genome.

References

1. Bartenhagen, C. & Dugas, M. RSVSim: an R/Bioconductor package for the simulation of structural variations. *Bioinformatics* **29**, 1679–1681 (2013).
2. Kapitonov, V. V. & Jurka, J. Molecular paleontology of transposable elements in the Drosophila melanogaster genome. *Proc Natl Acad Sci USA* **100**, 6569–6574 (2003).
3. Hoskins, R. A. *et al.* The Release 6 reference sequence of the Drosophila melanogaster genome. *Genome Res* **25**, 445–458 (2015).
4. Chakraborty, M. *et al.* Hidden genetic variation shapes the structure of functional elements in Drosophila. *Nat. Genet.* **50**, 20–25 (2018).
5. Hoyt, S. J. *et al.* From telomere to telomere: The transcriptional and epigenetic state of human repeat elements. *Science* **376**, eabk3112 (2022).
6. Nurk, S. *et al.* The complete sequence of a human genome. *Science* **376**, 44–53 (2022).
7. Song, J.-M. *et al.* Two gap-free reference genomes and a global view of the centromere architecture in rice. *Mol. Plant* **14**, 1757–1767 (2021).
8. Hosmani, P. S. *et al.* An improved de novo assembly and annotation of the tomato reference genome using single-molecule sequencing, Hi-C proximity ligation and optical maps. Preprint at https://doi.org/10.1101/767764.
9. Alonge, M. *et al.* Major Impacts of Widespread Structural Variation on Gene Expression and Crop Improvement in Tomato. *Cell* **182**, 145–161.e23 (2020).
10. Hufford, M. B. *et al.* De novo assembly, annotation, and comparative analysis of 26 diverse maize genomes. *Science* **373**, 655–662 (2021).
11. Liao, Y. *et al.* The 3D architecture of the pepper genome and its relationship to function and evolution. *Nat. Commun.* **13**, 3479 (2022).
12. Qin, C. *et al.* Whole-genome sequencing of cultivated and wild peppers provides insights into Capsicum domestication and specialization. *Proc. Natl. Acad. Sci. U. S. A.* **111**, 5135–5140 (2014).
13. Kiełbasa, S. M., Wan, R., Sato, K., Horton, P. & Frith, M. C. Adaptive seeds tame genomic sequence comparison. *Genome Res.* **21**, 487–493 (2011).
14. Marçais, G. *et al.* MUMmer4: A fast and versatile genome alignment system. *PLoS Comput. Biol.* **14**, e1005944 (2018).

15. Li, H. Minimap2: pairwise alignment for nucleotide sequences. *Bioinformatics* **34**, 3094–3100 (2018).
16. Song, B. *et al.* AnchorWave: Sensitive alignment of genomes with high sequence diversity, extensive structural polymorphism, and whole-genome duplication. *Proc. Natl. Acad. Sci. U. S. A.* **119**, (2022).
17. Lin, H.-N. & Hsu, W.-L. GSAlign: an efficient sequence alignment tool for intra-species genomes. *BMC Genomics* **21**, 182 (2020).
18. Nattestad, M. & Schatz, M. C. Assemblytics: a web analytics tool for the detection of variants from an assembly. *Bioinformatics* **32**, 3021–3023 (2016).
19. Goel, M., Sun, H., Jiao, W.-B. & Schneeberger, K. SyRI: finding genomic rearrangements and local sequence differences from whole-genome assemblies. *Genome Biol.* **20**, 277 (2019).
20. Heller, D. & Vingron, M. SVIM-asm: structural variant detection from haploid and diploid genome assemblies. *Bioinformatics* **36**, 5519–5521 (2021).
21. O'Donnell, S. & Fischer, G. MUM&Co: accurate detection of all SV types through whole-genome alignment. *Bioinformatics* **36**, 3242–3243 (2020).
